# Self-Healing Polymer Electrolytes for Next-Generation Lithium Batteries

**DOI:** 10.3390/polym15051145

**Published:** 2023-02-24

**Authors:** Anja Marinow, Zviadi Katcharava, Wolfgang H. Binder

**Affiliations:** Macromolecular Chemistry, Institute of Chemistry, Faculty of Natural Science II, Martin Luther University Halle-Wittenberg, Von-Danckelmann-Platz 4, 06120 Halle (Saale), Germany

**Keywords:** self-healing, electrolyte, polymer, lithium battery, hydrogen bonding, dynamic covalent bonds

## Abstract

The integration of polymer materials with self-healing features into advanced lithium batteries is a promising and attractive approach to mitigate degradation and, thus, improve the performance and reliability of batteries. Polymeric materials with an ability to autonomously repair themselves after damage may compensate for the mechanical rupture of an electrolyte, prevent the cracking and pulverization of electrodes or stabilize a solid electrolyte interface (SEI), thus prolonging the cycling lifetime of a battery while simultaneously tackling financial and safety issues. This paper comprehensively reviews various categories of self-healing polymer materials for application as electrolytes and adaptive coatings for electrodes in lithium-ion (LIBs) and lithium metal batteries (LMBs). We discuss the opportunities and current challenges in the development of self-healable polymeric materials for lithium batteries in terms of their synthesis, characterization and underlying self-healing mechanism, as well as performance, validation and optimization.

## 1. Introduction

The development of alternative power sources to traditional petrochemical energy in the pursuit of carbon neutrality is one of the biggest challenges nowadays. Advanced rechargeable batteries are one of the most promising strategies towards renewable power sources due to the fact of their high power, excellent power density, reusability, portability and relatively low cost [1,2,3,4]. Until now, lithium-ion-based batteries are dominating the secondary battery market, finding widespread use in different areas, such as e-mobility and in various portable electronic devices, such as smartphones, laptops, pacemakers, and watches [5,6,7]. However, our increased demand for energy-efficient battery technologies introduces compelling needs for enhanced energy and power densities, safety and cost savings, while simultaneously opening ethical questions including sustainability, repurposing or recycling [3,8,9]. Thus, there have been flourishing research activities in different directions to fulfill these prospective requirements. One of the favored approaches is the introduction of more diversity in energy storage systems by switching to more abundant resources, such as sodium (Na), potassium (K), calcium (Ca), magnesium (Mg), zinc (Zn) or aluminum (Al) [10,11,12,13,14,15,16]. However, the development of materials that enable the safe and efficient use of these other metal-based technologies is still in the early stages. Another promising approach is the development of advanced materials for the next-generation Li-ion and Li-metal-based batteries designed to enhance the performance and offset the drawbacks of conventional Li-ion batteries [6,17,18,19,20]. 

Polymers are crucial components of enhanced performance lithium batteries, e.g., as binders for electrodes and as a substrate for separators, electrolytes or package coatings [21,22,23]. However chemical reactions, as well as thermal changes, during a battery’s operation inevitably lead to structural changes, which may cause damage and degradation of the polymers applied as electrolytes, separators or binders on electrodes, thus limiting the practicality of batteries. Significant volume changes of electrode materials (especially in the case of a Si anode) during the charging/discharging of a lithium-ion battery (LIB) causes a pronounced fracture of the electrodes, a decrease in the interparticle contact followed by the loss of electrical insulation of electrode particles and electrode pulverization, thus resulting in significant irreversible capacity loss and poor cycling stability [21,24]. Regarding lithium metal batteries (LMBs), the volume changes of the Li-metal anode may lead to damage or even cracking of the artificial protective solid electrolyte interface (SEI) layer, subsequently leading to an inhomogeneous Li deposition and pronounced growth of Li dendrites. Distinct Li dendrites may penetrate through the separator membrane between the cathode and anode, inducing an internal short circuit (ISC) of the battery, followed by a thermal runway of the battery (smoke, fire or even explosion) [25,26]. Furthermore, LIBs with conventional liquid electrolytes generally suffer drawbacks from lithium dendrites [27], leakage and flammability of the liquid electrolytes and, therefrom, resulting in poor thermal stability [28]. Even if polymer electrolytes are used, they may break under the complex deformation during cycling, causing, in turn, contact between the electrodes and subsequent battery failure connected to severe safety issues [25]. Thus, significant efforts should be devoted to increasing the working lifetime of lithium batteries by improving their capability of sustaining internal and external stresses, e.g., cutting, breakage, volume expansion, gas release or heat [29,30].

The concept of self-healing is bioinspired and can be regarded as an attempt to mimic the ability of a living organism to heal and recover from minor injuries. For example, by looking at the superficial injuries in mammal organisms, we can observe that a vascular (e.g., bloodstream-supported) supply system helps to heal and restore mechanical damage via the blood-clotting cascade and subsequent tissue regeneration. Similarly, human skin can self-repair after injury, thus repeatedly regaining its functionality after healing. Hence, an important aspect of self-healing is the presence of a structure with an ability to respond dynamically to an external stimulus, enabling the restoration of the initial material properties. Due to the fact of their highly complex chain structure, polymers are ideally suitable to serve as materials with dynamic and, thus, self-healing properties [31,32]. Over the last twenty years, self-healing polymers and composites have emerged, offering an autonomous route to mitigate deterioration and prolong useful lifetime, finding their application in numerous fields, including energy storage devices [29,30,33,34,35] and flexible and stretchable electronics [36]. This usually involves the repair of mechanical cracks but may also be broadened to a wider range of features, generally prolonging the lifetime of a battery. 

Following the biomimetic approach and implementing self-healing features into advanced batteries is, to date, a prosperous research area. This paper aimed to provide a focused review of the research and development of polymeric self-healing materials in LIBs and LMBs, with a special focus on electrolytes and self-healing polymer binders for electrodes. The progress on lithium–sulfur and lithium–oxygen batteries was not within the scope of this review, and further information can be found elsewhere [30,35,37]. Furthermore, we focus on organic lithium batteries, since the development of self-healing hydrogels for aqueous lithium batteries has been recently reviewed [38,39,40,41]. Section 2 of this paper focuses on the general principles of self-healing polymeric materials, with an emphasis on the concepts suitable for application in batteries. Subsequently, the development of self-healing polymer electrolytes for LIBs and LMBs is presented and discussed in detail in Section 3. In Section 4, the development and electrochemical performance of self-healing polymers from the viewpoint of electrodes is presented. This flourishing research field reveals emerging perspectives and challenges, which are discussed in Section 5. 

## 2. Self-Healing Mechanism

A self-healing mechanism can be defined as the ability of a material to recover from damage, thus regaining its initial functionality, mimicking the repair processes found in living nature. Self-healing processes in polymer materials can occur with (i.e., nonautonomous) or without external stimuli (i.e., autonomous) and can generally be classified into two major groups: extrinsic self-healing via embedded healing agents and *intrinsic self-healing* via the reversible reformation of chemical bonds within the material [31,32,42,43]. Embedding of the healing agent in extrinsic self-healing systems can either be based on capsules or on vascular networks. Thereby, a liquid component is embedded either in microcapsules or microvessels during fabrication. After triggering (e.g., mechanical damage), the liquid component can flow to the damaged area to enable healing. Since no external stimuli is required, extrinsic self-healing is autonomous. The advantage of extrinsic self-healing is the ability to separate the active part of the material with the desired physical and chemical properties from the repairing one. However, in capsule-based self-healing systems, external damage causes the rapture of a capsule and the release of a healing agent, thus being connected to the exhaustion of the external chemical agent, leading to an irreversible self-healing process with a single healing event only. Moreover, for application in energy storage devices, the “activity” of the released healing agent, from an electrochemical point of view, plays an important role. Further, the self-healing efficiency is strongly dependent on the number of capsules in the matrix, indirectly influencing the properties and performance of the matrix. Vascular self-healing systems were originally developed to compensate for the shortcomings of the one-time self-healing of capsule-based systems [31,44]. In vascular self-healing systems, a healing agent is distributed in capillaries and hallow channels, forming an interconnected network and enabling a repeated self-healing process (up to ten times). However, similar to capsules, the main challenge and drawback is the compatibility of the matrix with the vascular networks and, subsequently, the inevitable negative impact on the original properties of the matrix material. Thus, regarding the development of self-healing polymers for next-generation lithium batteries, the focus is set on autonomous intrinsic self-healing processes [33,35]. 

**Figure 1:**
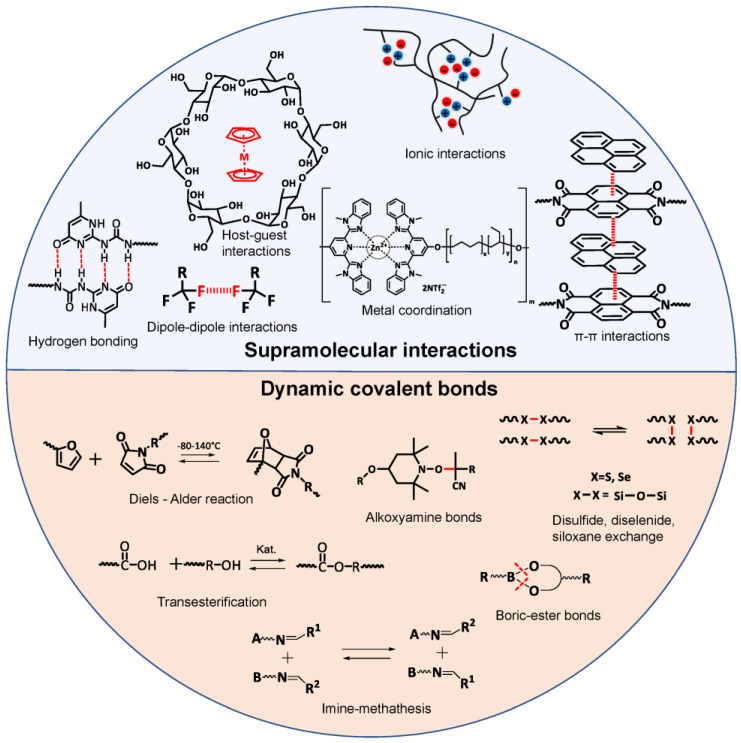
Chemical structures of the various supramolecular interactions and dynamic covalent bonds used for self-healing polymers.

Intrinsic self-healing is an autonomous self-healing process that depends on tailoring the dynamic bonding interaction at the molecular level or framing an interplay between the diffusion and entanglement of polymer chains. Intrinsic self-healing in polymers can be achieved either via supramolecular interactions [42] or via dynamic covalent bonds [45,46] (Figure 1). Supramolecular chemistry is based on reversible noncovalent bonds, which primarily break upon mechanical stressing/damage, leaving behind “sticky” functionalities at the freshly generated interface comprising multiple unbounded supramolecular bonds, which in turn can recombine to restructure (“heal”) the material. The most important factors for using supramolecular interactions for self-healing are their strength (resulting either in a stronger or weaker supramolecular network) and the dynamics of the self-healing processes (reestablishing the broken bonds after damage) [47,48]. Supramolecular interactions include hydrogen bonds [24,48,49,50], metal–ligand coordination [51], electrostatic (ionic) interactions [52], host–guest interactions [53], π–π stacking [54,55], dipole–dipole interactions [56] and van der Waals interactions [32]. Self-healing via dynamic covalent bonds can generally be achieved by shifting the equilibrium from an energetically higher to an energetically lower state. The basic principle of self-healing via dynamic covalent bonds is the opening and closing of the bonding sites, which leads to an equilibrium. The control of the equilibrium by triggering the formation or cleavage of covalent bonds can be achieved by different external stimuli, such as irradiation, pH-value, moisture, catalytic activity and temperature. Heat is the most used trigger, because it is easily tunable and relevant for different applications [34,46,57]. Dynamic covalent bonds used in self-healing materials include Diels–Alder reaction [58,59], disulfide [60], diselenide [61] or siloxane [62], exchange reactions, boric–ester bonds [38], imine bonds [63], transesterification reactions [64] and alkoxyamine bonds [65]. The reversibility is the major advantage of intrinsic self-healing processes, enabling the perpetual renovation of the material. Additionally, no additional weight from an electrochemically inactive material (e.g., capsules or microvessels) needs to be incorporated in the energy storage system. One of the limiting factors is the relatively slow kinetics of the bond re-organization, usually making intrinsic self-healing slower than the extrinsic one. In addition, in order to undergo autonomous self-healing via the reversible iterative reformation of the bonds, the material needs to permit sufficient mobility, thus restricting the material choice to polymers above the glass transition temperature (T_g_). 

In the next section, we discuss in detail the application of the abovementioned intrinsic self-healing polymeric materials as electrolytes or adaptive binders for electrodes in lithium batteries. 

## 3. Self-Healing Electrolytes for Lithium Batteries

### 3.1. Opportunities and Challenges for Electrolytes 

**Figure 2:**
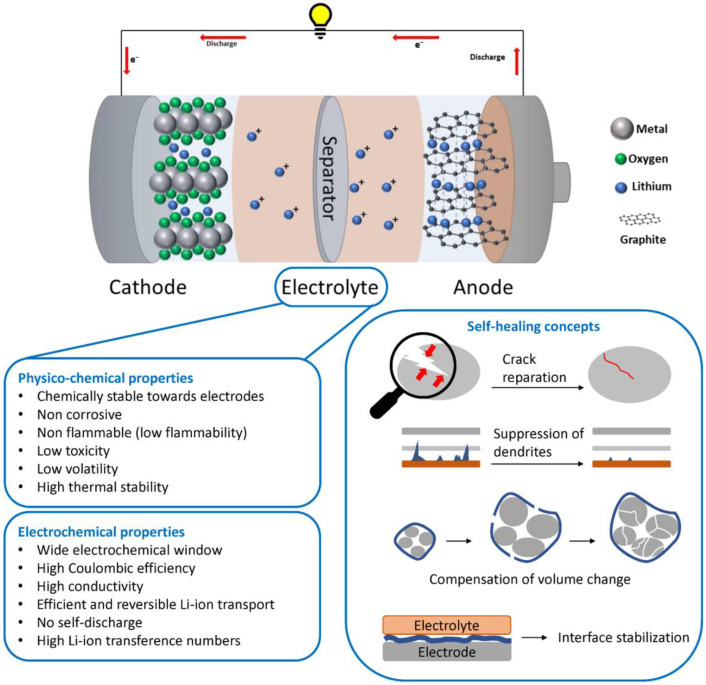
Schematic illustration of a Li-ion battery. The requirements of electrolytes in lithium-based batteries (**left**) and the different roles of self-healing polymers in lithium batteries (**right**).

The basic working principle of the LiB is schematically presented in Figure 2. Li-ion rechargeable batteries consist of two electrodes, anode and cathode, immersed in an electrolyte and separated by a polymer membrane (i.e., separator) in order to electrically isolate the electrodes from each other. Single lithium ions are transferred back and forth between the anode and the cathode through the electrolyte during charging and discharging. Currently, most intercalation reactions are widely applied in commercial Li-ion batteries for the storage of lithium ions, both in anodes and in cathodes [20]. The charge transport is based on solvated Li^+^ ions and their aggregates with corresponding anions, while solvation/de-solvation processes and corresponding electrochemical reactions govern the charge transfer at interfaces and through interphases (i.e., solid electrolyte interphase (SEI) and cathode electrolyte interphase (CEI)) during operation [5]. The choice of the electrolyte plays a very important role. A successful electrolyte is required to play multiple critical roles in an electrochemical cell: (1) it should isolate the electron and ion transport pathways in the cell; (2) it should promote ion-pair dissociation and selectively facilitate the transport of the active ionic species (e.g., Li^+^ ions in a lithium battery); (3) it must penetrate and wet the porous, chemically heterogeneous hybrid materials that constitute the electrodes and separator; (4) it should not leak, combust or vaporize during cell storage or operation; (5) it should be chemically robust in the presence of the electrodes and their redox products. Finally, (6) the electrolyte itself must be stable in the normal operating voltage range for the electrochemical cell (3.5–5 V, depending on the cell system). The requirements of the electrolyte are summarized in Figure 2. 

Currently, in commercially available LiBs, predominantly liquid aprotic electrolytes based on different carbonates such as dimethyl carbonate are used. Such electrolytes are characterized by high ionic conductivity (up to 10^−1^ S cm^−1^) and low costs. However, they exhibit severe disadvantages, such as flammability and leakage. Significant work has been invested in the development of new electrolytes for high-energy density batteries over the past years [11,66,67].

One of the approaches to solving the drawbacks connected with conventional liquid electrolytes (leachability, safety issues, etc.) is the development of solid polymer electrolytes (SPEs), mostly consisting of Li-salts dispersed in a polymer matrix. SPEs significantly increase the mechanical integrity and thermal stability of electrolytes, successfully solving problematic safety issues connected with the leachability of flammable liquid electrolytes [2,11,66,68]. Furthermore, SPEs allow for the development of light and low-cost materials, simultaneously being able to overcome different obstacles, such as lithium dendrite growth and uncontrollable and undesired side reactions or thermal runaway [69]. However, one of the main drawbacks of polymer electrolytes is their significantly lower conductivity (10^−6^–10^−8^ S cm^−1^). Furthermore, SPEs tend to dissipate and, thus, slow down during battery charging/discharging cycles, leading to direct contact between electrodes, subsequently resulting in catastrophic failure of the batteries and serious safety issues. 

Thus, it is of great importance to develop materials capable of enduring mechanical stress during charging/discharging processes (lithiation/delithiation) to improve the overall lifetime and performance of the next-generation lithium batteries. The incorporation of intrinsic self-healing features in polymer-based electrolytes is a promising approach to overcome the previously mentioned safety issues. In general, self-healing polymer electrolytes for lithium batteries can be classified into three types according to their composition: (1) solid polymer electrolytes (SPEs), (2) gel polymer electrolytes (GPEs) and (3) composite polymer electrolytes (CPEs). The general synthetic concept, underlying self-healing mechanism and conditions, as well as the reported electrochemical performance of the developed self-healing polymer electrolytes, are summarized in Table 1 for comparison and are discussed in the following sections in detail.

### 3.2. Self-Healing Solid Polymer Electrolytes (SPEs)

An SPE consists of lithium salt dissolved in a polymer matrix without the addition of a solvent or a plasticizer. Poly (ethylene oxide) (PEO) is the most used SPE, and the mechanism of Li-ion transport has been extensively studied. Flexible ethylene oxide segments and ether oxygen atoms were found to be good donors for Li^+^ transport, enabling the hopping of an ion from one oxygen coordination site to another. Additionally, the segmental motion of polymer chains further promote ion transport. However, at room temperature, PEO is a crystalline polymer, consequently exhibiting low conductivity. The major drawback of PEO-based electrolytes is their modest ionic conductivity (i.e., <10^−5^ S cm^−1^ at RT), which obligates the battery to operate above 60 °C, where it reaches acceptable values (at least 10^−3^ S cm^−1^) owing to the phase transition from a crystalline to highly conductive amorphous phase [66,107]. Thus, it is not surprising that the incorporation of self-healing features into PEO-based polymers is an interesting approach. In general, the integration of PEO chains into a self-healing polymer matrix (e.g., via copolymerization) may lead to a decrease in the crystallinity by disturbing the regular packaging of PEO chains, resulting in self-healable SPEs with high ionic conductivity. 

Among supramolecular self-healing materials, hydrogen bonding (HB) plays an important role. Although HBs belong to weaker interactions in the range of 5 to 40 kJ mol^−1^, the presence of hydrogen bonding strongly influences the viscoelasticity and degree of phase separation, as well as the degree of crystallinity of the materials due to the fact of their directionality and affinity. The dynamics of the opening and closing of the supramolecular networks (thus, the efficiency of the self-healing), as well as the mechanical integrity of the material, can be tuned by the concentration, type and strength of the hydrogen bonds together with the rigidity of the polymer backbone and the position of the hydrogen-bonding supramolecular entity along the polymer chain [31,42,108]. Thus, it is not surprising that the introduction of HBs into polymeric materials leads to the improvement of mechanical properties, shape-memory, stimuli responsive or self-healing features [47,109,110,111,112].

**Figure 3:**
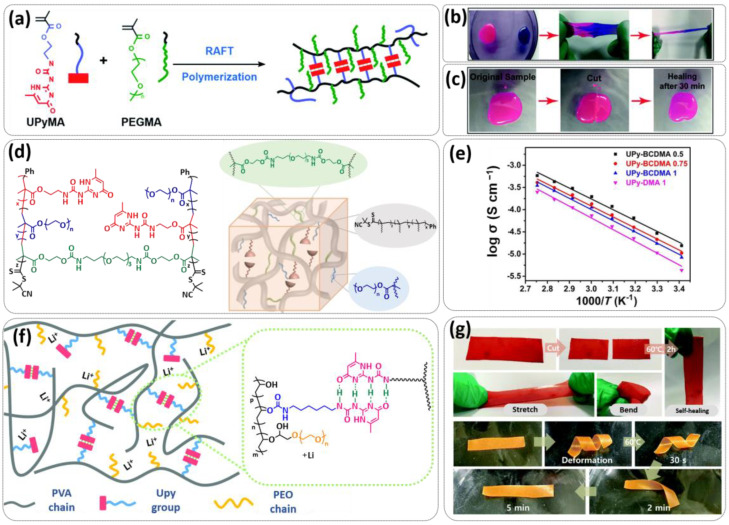
(**a**) Schematic illustration of the synthesis of brush-like copolymer starting from UPyMA and PEGMA monomers. (**b**) Optical images showing the self-healing behavior of PEG5-UPy at ambient conditions (above) and (**c**) at 60°C (below). Reproduced from [79] with permission from the Royal Society of Chemistry. (**d**) Schematic illustration of the dual-network of self-healing UPy-BCDMA. (**e**) The temperature dependence of the ionic conductivity for UPy-BCDMA copolymer electrolytes. Reproduced with permission from [71]. Copyright © 2018 Wiley-VCH Verlag GmbH & Co. KGaA, Weinheim. (**f**) Schematic illustration of the preparation process of PVA-UPy-PEG. (**g**) Optical images of the self-healing (up) and shape-memory (down) behavior of PVA-UPy-PEG. Reproduced from [76] with permission from the Royal Society of Chemistry.

In 2018, Zhou et al. successfully introduced a quadrupole hydrogen bonding supramolecular self-healing moiety into a PEO-based SPE (Figure 3a; Table 1, entry 12) [79]. The self-healing SPE was prepared via the reversible addition-fragmentation chain transfer (RAFT) polymerization of a monomer containing a quadrupole hydrogen bonding ureidopyrimidinone (UPy) moiety (2-(3-(6-methyl-4-oxo-1,4-dihydropyrimidin-2-yl)ureido)ethyl methacrylate) (UPyMA) and poly(ethylene glycol) methyl ether methacrylate (PEGMA). PEG side chains facilitate good ionic conductivity, whereas the dynamic supramolecular crosslinking of UPy groups provides a framework for flexible, freestanding and self-healing SPEs. The ionic conductivity of PEG5-UPy (PEGMA:UPyMA = 5:1) reached 2.1 × 10^−5^ S cm^−1^ at 30 °C and even 1.1 × 10^−4^ S cm^−1^ at a working temperature of 60 °C. As can be seen in Figure 3b,c PEG5-UPy could be healed after cutting at 30 min at 60 °C (or 2 h at RT) and was easily stretched with more than a 2000% strain. In addition, the LiFePO_4_/PEG5-UPy/Li battery exhibited an initial capacity of 157 mAh g^−1^ and maintained a very low and stable voltage polarization after cycling over 500 h while sustaining a stable cycling performance. 

The same working group further extended this concept by incorporating the third monomer polyethylene glycol-bis-carbamate dimethacrylate (PEGBCDMA) as a chemical crosslinker (see Figure 3d; Table 1, entry 2) developing a dual-network structure which is crosslinked by quadruple hydrogen bonding and chemical bonds [71]. The formation of the second network via the chemical crosslinking of PEGBCDMA guarantees the dimensional stability and good mechanical properties of the polymer electrolyte. Although the SPE with a higher fraction of the chemical crosslinker PEGBCDMA (UPy-BCDMA 1, PEGMA:UPyMA:PEGBCDMA = 5:1:1) exhibited a lower ion conductivity (1.07 × 10^−5^ S cm^−1^ at 30 °C, Figure 3e) compared to the SPE with the lower fraction of PEGBCDMA (UPy-BCDMA 0.5, PEGMA:UPyMA:PEGBCDMA = 5:1:0.5, ion conductivity 2.93 × 10^−5^ S cm^−1^ at 30 °C), its mechanical integrity made it more suitable for evaluation as an SPE for LMBs. The tensile stress of the original UPy-BCDMA 1 was 0.13 MPa, and the strength of the healed sample after 2 h at 60 °C was 0.11 MPa (84 %). The LiFePO_4_/UPy-BCDMA 1/Li battery exhibited an initial capacity of 137.9 mAh g^−1^, with a coulombic efficiency of 99% at a current density of 0.1 C. The discharge capacity was retained at 128 mAh g^−1^ after 120 cycles, and the capacity retention of the battery was over 90%. Furthermore, the pouch cell fabricated with UPy-BCDMA 1 could power an LED device even under deformation or folding conditions.

Another interesting approach to use a quadrupole hydrogen bonding UPy self-healing moiety for the preparation of SPE was presented a year later by Jo et al. [76]. They fabricated self-healing and shape-memory SPEs based on poly(vinyl alcohol) (PVA) with ureidopyrimidinone (UPy) and poly(ethylene glycol) (PEG) units (PVA-Upy-PEG) (Figure 3f; Table 1, entry 8). The SPEs were prepared in a polyaddition reaction of different chain length epoxide functionalized-PEG and 2(6-isocyanatohexylaminocarbonylamino)-6-methyl-4[1*H*]-pyrimidinone (UPy-NCO) with the hydroxyl groups of PVA. PVA features numerous properties that make it a promising candidate as a polymer matrix for SPEs, such as good thermal and mechanical properties, nontoxic nature, relatively high dielectric constant and a good film-forming property. The PVA-UPy-PEG750 showed a high ionic conductivity of 1.51 × 10^−4^ S cm^−1^ with an EO/Li^+^ ratio of 11:1 at 60 °C, wide electrochemical window (5.0 V vs. Li/Li^+^) and a lithium-ion transference number of *t*_Li_^+^ = 0.34. Furthermore, the tensile stress of PVA-UPy-PEG750 could reach 2.56 MPa, which is significantly higher compared to other PEG-UPy polymers (~40 kPa). The self-healing and shape-memory behavior of PVA-UPy-PEG750 is presented in Figure 3g. After cutting, the PVA-UPy-PEG750 film successfully healed after 2 h at 60 °C. After heating to 60 °C, the SPE could be rolled and frozen in liquid nitrogen to form a spiral-like shape. Notably, the PVA-UPy-PEG750 could transform from a temporary shape to a permanent shape under a heat stimulus (60 °C). The temporary shape was controlled via PVA main chains and PEG side chains that acted as a reversible phase, whereas the permanent shape was regulated by a physically crosslinked network formed via quadruple hydrogen bonding. The Li/PVA-UPy-PEG750/LiFePO_4_ cell exhibited a higher initial discharge capacity of 145 mA h g^−1^ while maintaining a discharge capacity of 117 mA h g^−1^ after 150 cycles and a coulombic efficiency of 99% with 0.1C at 60 °C. 

**Figure 4:**
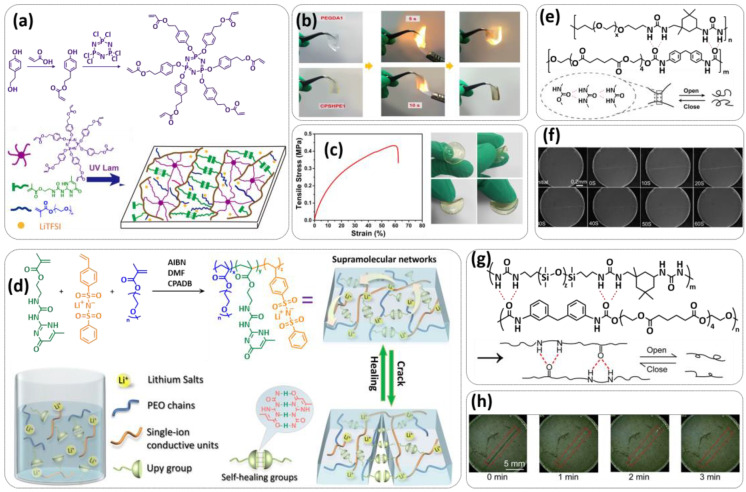
(**a**) Schematic illustration of the synthesis route of HCP followed by the fabrication of CPSHPEs via photopolymerization under UV irradiation. (**b**) States of the membrane without (PEGDA1) and with HCP (CPSHPE1) at different burning times. (**c**) Mechanical properties of the CPSHPE1 (intercept: folding of the CPSHPE1). Reproduced with permission from [72]. Copyright © 2020, American Chemical Society. (**d**) Schematic illustration for the preparation process of SIPE membranes. Reproduced with permission from [90]. Copyright © 2021, American Chemical Society. (**e**) Supramolecular structure and dynamic hydrogen bonding in SHSPE. (**f**) The self-healing process of SHPE after cutting. Reproduced with permission from [70]. Copyright © 2019, Wiley-VCH Verlag GmbH & Co. KGaA, Weinheim. (**g**) Molecular structure and dynamic hydrogen bonding of SHSPE_2 and (**h**) optical images of its self-healing at room temperature. Reproduced from [86], with permission from the Royal Society of Chemistry.

In order to improve the fire resistance of the self-healing PEG-UPy SPEs, the same working group presented cyclophosphazene-based self-healing polymer electrolytes (CPSHPE) fabricated via the photoinitiated radical copolymerization of hexa(4-ethyl acrylate phenoxy) cyclotriphosphazene (HCP), (2-(3-(6-methyl-4-oxo-1,4-dihydropyrimidin-2-yl)ureido)ethyl methacrylate) (UPyMA) and poly(ethylene glycol) methyl ether methacrylate (PEGMA) (see Figure 4a; Table 1, entry 3) [72]. The crosslinking structure formed by HCP is, on the one hand, effectively enhancing the mechanical strength of the polymer electrolyte, and on the other hand, the cyclotriphosphazene as the core rich in N = P conjugated rings is responsible for the excellent flame-retardant properties (Figure 4b). Furthermore, prepared CPSHPE is foldable and shows high tensile strength of 0.45 MPa and an elongation of 60% (Figure 4c). Quadrupole hydrogen bond moieties of the UPy group enable the formation of supramolecular networks and self-healing behavior (3 h at 60 °C). The ionic conductivity of the CPSHPE reaches 3.7 × 10^−4^ S cm^−1^ at 60 °C, which can meet the requirement for practical application in lithium batteries. The specific capacity of the assembled LMB cell Li/CPSHPE1/LiFePO_4_ was 130 mhA g^−1^, with a Coulombic efficiency of 99.1% at 0.1C. Due to the self-healing ability combined with high mechanical integrity, CPSHPE additionally enables the efficient suppression of lithium-dendrite formation, resulting in the long-term overvoltage stability of up to 200 h. The same research group further successfully applied a quadrupole hydrogen bonding UPy moiety for the fabrication of self-healing single-ion conducting polymer electrolytes (SIPEs), which should reduce the concentration polarization, thus leading to the suppression of the formation of lithium dendrites and healing of the cracks when applied as SPEs in LMBs [90]. The SIPEs were synthesized via reversible addition–fragmentation chain transfer (RAFT) copolymerization of lithium 4-styrenesulfonyl(phenylsulfonyl)imide (SSPSILi), 2-(3-(6-methyl-4-oxo-1,4-dihydropyrimidin-2-yl)ureido)ethyl methacrylate (UPyMA) and poly(ethylene glycol) methyl ether methacrylate (PEGMA) in the absence of additional lithium salts (Figure 4d; Table 1, entry 23). The best performance was achieved with SIPE-5 containing the highest fraction of lithium conducting SSPSILi (PEGMA:UPyMA:SSPSILi = 5:1:5), including a good thermal stability up to 278.4 °C, comparatively high ionic conductivity up to 1.40 × 10^−5^ S cm^−1^ at 60 °C, high lithium-ion transference number (t_Li_^+^ = 0.89) and good compatibility with the lithium metal anode. Additionally, after cutting, the SIPE-5 membrane could be successfully healed after 12 h at 60 °C, exhibiting a robustness of 0.42 MPa and an elongation of 285% at 60 °C. 

Until now, we discussed almost exclusively the examples of self-healing SPEs based on the quadrupole hydrogen bonding networks of UPy moieties; however, other types of hydrogen bonding can also be applied for the preparation of SPEs for lithium batteries. For example, Wu et al. [70] and Zhang et al. [86] used a similar approach for the preparation of self-healable SPEs via multiple molecule/intermolecular dynamic reversible hydrogen bonds. In 2019, Wu et al. [70] fabricated a self-healing polymer SHSPE featuring rapid self-healing after cutting within 60 s at room temperature (Figure 4e,f; Table 1, entry 1) via simple polycondensation reaction. Amino-terminated poly(ethylene glycol) (NH_2_-PEG-NH_2_) was chosen as the supramolecular backbone based on its dynamic hydrogen bonding which, on the one hand, leads to self-healing features and, on the other had, takes an active part in Li-ion transport over PEG chains. Thermoplastic polyurethane (TPU) was used as a physical crosslinker to improve the mechanical strength of SHSPE, which was crosslinked to the PEG-based supramolecular scaffold through intramolecular hydrogen bonding between the urea groups and ester groups. Thus, the dynamic and reversible intermolecular or intramolecular hydrogen bonding between a large number of present urea and ester groups led to a fast self-healing response at the molecular level. Optimized SHSPE after the addition of 2 M lithium salt (LiClO_4_) could reach an ion conductivity of 1.9 × 10^−4^ S cm^−1^, showing an electrochemical stability up to 5 V (vs. Li^+^/Li). Lithium storage capacities of approximately 147.9 mAh g^−1^ (for the Li|SHSPE|LFP battery) and 170.6 mAh g^−1^ (for the Li|SHSPE|NCM battery) at 0.1 C, respectively, were reached. In the subsequent recent work of Zhang et al. [86], a similar procedure was used; however, the amino-terminated PEG was replaced with amino-terminated polydimethylsiloxane (N-PDMS) as a dynamic hydrogen-bonding skeleton (Figure 4g; Table 1, entry 19). Due to the good compatibility of the components, the obtained SHSPE_2 exhibited a smooth surface and good film-forming properties. The prepared SHSPE_2 with a ratio of N-PDMS to TPU of 20:1 and a 2 M lithium salt concentration (LiTFSI) reached 2.4 × 10^−4^ S cm^−1^, whereas the self-healing was reached in 3 min at room temperature. 

In addition to hydrogen bonds, other types of supramolecular interactions can also be used for the preparation of self-healing SPEs, for example, ionic interactions. So-called ionic liquids (ILs), organic salts with a melting point below 100 °C, is a class of purely ionic compounds with a series of favorable properties, such as low vapor pressure, nonflammability, high conductivity (~10^−2^ S cm^−1^), high thermal stability and large electrochemical window (4.5–5 V). Due to the fact of their unique properties, ILs have been extensively studied for application in energy storage devices, among others, and also as a substitute for conventional organic liquid electrolytes [8,113,114]. ILs may solve some of the problems connected with conventional electrolytes, such as safety issues due to the fact of flammability, or even provide a positive effect on the formation of the stable SEI layer. However, they are still liquid at room temperature and, thus, still prone to leaching and unsuitable for the incorporation of self-healing features. There are, in general, two possibilities to increase the mechanical integrity of ILs and introduce the self-healing function: (1) preparation of gel materials, so-called ionogels, by embedding ILs into polymeric materials (either via chemical crosslinking or via physical mixing) [115,116,117] or (2) using ILs as monomers for the preparation of so-called polymeric ionic liquids (PILs) [68,118]. The properties and applications of self-healing ionogels will be discussed in the next section; here, we focus on IL-based self-healing SPEs. 

**Figure 5:**
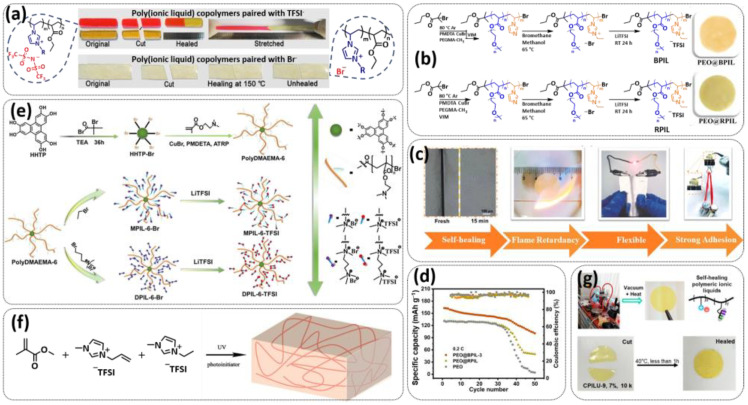
(**a**) Self-healing behavior of PILs containing TFSI^−^ and Br^−^ anions. Reproduced with permission from [52]. Copyright © 2018, American Chemical Society. (**b**) Synthetic steps towards block-copolymeric IL (BPIL) and its random analog (RPIL). (**c**) Properties of PEO@BPIL. (**d**) Cyclic tests of LiFePO_4_/SPEs/Li cells at 0.2C (60 °C). Reproduced with permission from [89]. Copyright © 2022, American Chemical Society. (**e**) Synthetic approach for the six-armed MPIL-6 and DPIL-6. Reproduced with permission from [77]. Copyright © 2021 Elsevier B.V. All rights reserved. (**f**) Schematic illustration of the synthesis of EMIMTFSI/P(MMA-co-AMIMTFSI) polymer electrolyte. Reproduced with permission from [81]. Copyright © 2022 Elsevier B.V. All rights reserved. (**g**) Visualized self-healing of CPILU. Reproduced from [49].

In 2018, Guo et al. reported for the first time a self-healing PIL [52]. A series of PILs was prepared by the statistical radical copolymerization of IL-monomer 1-(2-ethoxyethyl)-3-vinylimidazolium bromide and an ethyl acrylate monomer (EA), and the alternation of the monomer ratio and subsequent anion exchange led to different PILs (see Figure 5a; Table 1, entry 9). The choice of an anion plays an important role, since the self-healing behavior is governed by the electrostatic interaction between cations of the polymer chain and the corresponding anions. The bulky TFSI anion (bis(trifluoromethanesulfonyl)imide) leads to weaker electrostatic interactions, allowing greater mobility of the polymer chains, thus enabling the self-healing. The obtained PIL- films were flexible, exhibiting low strength but high deformability (tensile strength and Young’s modulus of 0.21 and 0.56 MPa, respectively, with a strain at break as high as 877%). Self-healing was achieved after 7.5 h at 55 °C. However, the ion conductivity of the PILs was relatively low (1.6 × 10^−7^ S cm^−1^ at RT), and the electrochemical performance of the PILs was not further investigated. Recently, Zhu et al. reported flexible self-healing PIL-based SPEs for lithium batteries [89]. A self-healing solid-state electrolyte (PEO@BPIL) was fabricated by the incorporation of an imidazolium-based polymerized ionic liquid–(poly(ethylene glycol) monomethacrylate (PEGMA)) block polymer into PEO. The incorporation of PIL-block copolymer into the PEO matrix reduced the crystallinity of PEO, while simultaneously an exceptional orderly and disordered microphase separation structure was introduced in PEO@BPIL, which formed “fast tracks” for Li-ion migration (Figure 5b; Table 1, entry 22). Polymeric ionic liquid block copolymers (BPILs) were prepared via living atom transfer radical polymerization (ATRP). The PEO@BPIL possessed a wide electrochemical window (5.0 V), high ionic conductivity (2.2 × 10^−4^ S cm^−1^ at 60 °C) and very good lithium transference number (t_Li_^+^ = 0.63). Additionally, the PEO@BPIL exhibited favorable properties, such as flexibility, strong adhesion and flame retardancy (Figure 5c), while internal self-repair was achieved in less than 30 min at 60 °C. The assembled Li/PEO@BPIL-3-SPE/LiFePO_4_ cell could release an outstanding specific capacity of 163 mAh g^−1^ at 0.2 C; additionally, after 50 cycles, the capacity retention rate reached approximately 81% (Figure 5d). 

In order to increase the ion conductivity of the PIL-based SPEs and additionally to introduce a significant amount of ionic interactions capable of generating enhanced self-healing abilities. Li and coworkers recently prepared a series of 6-armed mono- and dicationic PILs (Figure 5e; Table 1, entry 10) [77]. Six-armed poly(N,N,N-trimethyl-N-((2-(dimethylamino)ethyl methacrylate)-7-propyl)-ammonium dual bis(trifluoromethanesulfonyl) imides) (dicationic polymeric ionic liquids (DPILs)) were prepared by ATRP following the synthetic procedure shown in Figure 5e and subsequently used for the preparation of SPEs by mixing with ionic liquid 1-ethyl-3-methylimidazolium bis(trifluoromethanesulfonyl) imide (EMIMTFSI) and corresponding LiTFSI salt. The SPE prepared with a dicationic six-armed PIL (DPIL-6-SPE) significantly strengthened the ionic attractions and Van der Waals’ forces among the polymer segments and exhibited a prominent self-healing ability (recovery time < 2 h, 25 ℃) and a superior adhesion strength (weight loading > 200 g) towards lithium electrodes, signifying an excellent interfacial compatibility. The multiple cationic center in DPIL-6-SPE facilitates the dissociation of lithium salt and liberates more Li^+^ from trapping with TFSI-, thereby resulting in a superior ionic conductivity (over 10^−5^ S cm^−1^ at room temperature) and lithium-ion transference number (t_Li_+ = 0.46). Additionally, DPIL-6-SPE showed a highly stretchable (extensibility > 1500% and stress > 490 kPa), nonflammable and notch-insensitive behavior. The assembled LiFePO_4_/DPIL-6-SPE/Li battery could deliver a high discharge capacity of 152.6 mAh g^−1^ at 0.1C, and its capacity retention rate reached 94% after 50 cycles with a Coulombic efficiency of 96%. 

In 2022, Guo et al. presented a solvent-free green synthesis of nonflammable and self-healing polymer film electrolytes for LMBs [81]. Methyl methacrylate (MMA) and 1-allyl-3-methylimidazolium bis(trifluoromethanesulfonyl)imide (AMIMTFSI) were copolymerized via free radical polymerization initiated by UV light and used as the membrane-forming backbone containing ion transport channels. 1-Ethyl-3-methylimidazolium bis((trifluoromethyl)sulfonyl)imide (EMIMTFSI) was added to the membrane to further enhance the ionic conductivity (Figure 5f; Table 1, entry 14). Due to the existence of IL monomer units and free IL in the prepared electrolytes, the fast interaction of the ionic bonds a faster self-healing ability of the films, and the flame retardancy of the ionic liquids was fully maintained after polymerization. The prepared electrolytes showed good flame retardance properties, good mechanical properties (tensile rate ∼ 400%) and a high thermal decomposition temperature (>260 °C). The SPE prepared with a 40% fraction of free IL reached an ionic conductivity of 1.9 × 10^−4^ S cm^−1^ at room temperature, a decomposition voltage of 4.6 V (vs. Li/Li^+^) and could achieve an initial discharge capacity of 134.9 mAh g^−1^ with a capacity retention of 96.4% after 90 cycles at 0.1C for LiFePO_4_/Li half-cell at 25 °C. However, it should be mentioned that in the last two examples, a significant fraction of free IL (EMIMTFSI) was used. Thus, the term “solid polymer electrolyte” is questionable, and these materials should better be categorized under gel polymer electrolytes, or ionogels. 

Recently, we applied reversible addition–fragmentation chain-transfer (RAFT) polymerization for the preparation of quadrupolar-hydrogen-bonded self-healing copolymeric ionic liquids (CPILUs) (Table 1, entry 6) [49]. Due to the fact that pyrrolidinium-based ionic liquids generally have a higher electrochemical stability [119], we chose an acrylate monomer bearing pyrrolidinium cation, whereas acrylate-Upy monomer was used to introduce self-healing features. The obtained copolymers exhibited excellent thermal stability up to 360 °C and efficient self-healing in less than 1 h at 40 °C (Figure 5g). The developed CPILUs, when mixed with Li-salts, may be potential candidates for application as SPEs, and further investigations are currently underway. 

**Figure 6:**
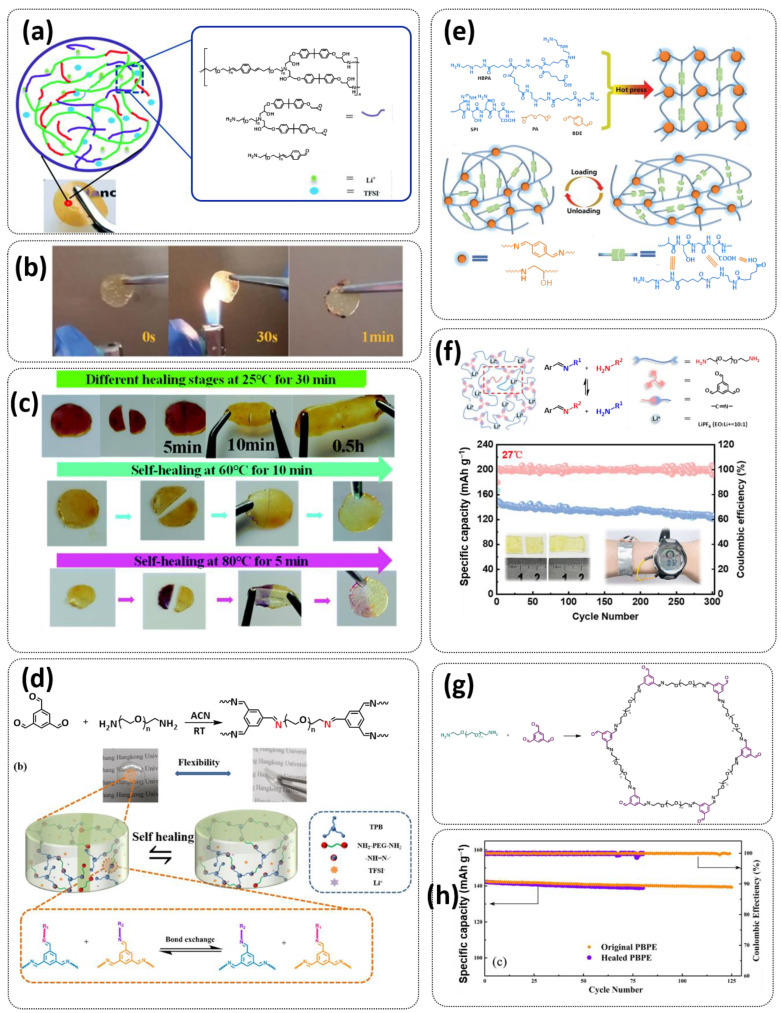
(**a**) Schematic illustration of the ShSPE-3 network. (**b**) Flammability test of ShSPE-3. (**c**) Self-healing behavior of ShSPE-3 at different temperatures. Reproduced from [78] with permission from the Royal Society of Chemistry. (**d**) Synthesis of crosslinked polymer and schematic diagram of the self-healing process of shCLSPE-3400. Reproduced with permission from [85]. Copyright © 2022, Society of Industrial Chemistry. (**e**) Preparation of the dynamic covalent networks of SPI-based vitrimer and schematic illustration of the energy dissipation mechanism through reversible breaking and reformation of hydrogen bonds under deformation. Reproduced with permission from [80]. Copyright © 2022 The Authors. Advanced Science published by Wiley-VCH GmbH. (**f**) Supramolecular structure and self-healing mechanism based on dynamic imine bonds for the SHSPE. The long-term cycling performance of the Li|SHSPE|LiFePO4 cell at 0.1C. Intercepts: self-healing behavior and flexible Li|SHSPE|LiFePO4 pouch cell. Reproduced with permission from [73]. Copyright © 2021, American Chemical Society. (**g**) Synthesis of PBPE. (**h**) Cycle performances of LFP/original PBPE/Li cell and LFP/healed BPE/Li cell at a 1C rate. Reproduced with permission from [74]. Copyright © 2021 Elsevier B.V. All rights reserved.

Until now, we discussed the self-healing SPEs based on the supramolecular interactions, however also self-healing polymeric materials based on dynamic covalent bonds are widely studied as SPEs for lithium batteries. More recently, several approaches towards introduction of imine dynamic covalent bonds into self-healing polymers are presented. Cao et al. fabricated a transparent, flexible and fire-resistant self-healing solid polymer electrolyte (ShSPE) in a simple condensation crosslinking reaction between diglycidyl ether of bisphenol A (DGEBA), terephthalaldehyde (TPA) and polyoxyethylenebis(amine) (NH_2_–PEG–NH_2_) in acetonitrile (Figure 6a–c; Table 1, entry 11) [78]. Thereby, dynamic imine bonds were formed through a simple Schiff base reaction between polyoxyethylenebis(amine) and terephthalaldehyde, whereas adding diglycidyl ether of bisphenol A improved the flexibility and stretchability of the polymer electrolyte. In an optimized polymer electrolyte, the molar ratio of TPA, DGEBA and NH_2_–PEG–NH_2_ of 1:2:3 (ShSPE-3) was used, while the EO/Li^+^ ratio was kept at 16:1 with the addition of LiTFSI. The ShSPE-3 showed the highest ionic conductivity of 1.67 × 10^−4^ S cm^−1^ at 60 °C and could be fully recovered after 30 min at RT, 10 min at 60 °C or only 5 min at 80 °C. Additionally, the interfacial stability of the ShSPE-3 was promoted and the LiFePO_4_/Li cell with the electrolyte membrane exhibited a good cycling performance and an initial discharge capacity of 141.3 mA h g^−1^ at 0.1C. The Schiff base reaction was also used for the preparation of the crosslinked network between polyoxyethylenebis(amine) (H_2_N-PEG-NH_2_) and 1,3,5-benzenetricarboxaldehyde, as presented in Figure 6d (Table 1, entry 18) [85]. It was found that the shCLSPE-3400 electrolyte with an appropriate PEG chain (M_n_ = 3400 Da) simultaneously exhibited good thermal stability, excellent self-healing ability (10 min at RT) and high mechanical strength due to the introduction of a crosslinked network structure and dynamic reversible bonds in the electrolyte. An ionic conductivity of 4.8 × 10^−4^ S cm^−1^, wide electrochemical stability window (5.1 V) and good lithium-ion transfer number (*t*_Li+_ = 0.31) with an ethylene oxide/Li^+^ ratio of 20:1 at 25 °C could be achieved. The Li|shCLSPE-3400|LiFePO_4_ battery showed good cycling stability, whereas the discharge capacity remained at 125.1 mAh g^−1^, with a coulombic efficiency of 97.9% after 100 cycles. 

Vitrimeric recyclable and self-healing solid polymer electrolytes (SPEs) with a soy protein isolate (SPI)-based imine bond dynamic network was presented last year by Gu et al. (Figure 6e; Table 1, entry 13) [80]. In general, vitrimers are regarded as polymeric materials composed of dynamic covalent networks that can change their topology by thermally activated bond-exchange reactions. The SPI-based vitrimer with a dynamically crosslinked polyimine network was prepared using commercially available starting material SPI and hyperbranched polyamide (HBPA). The HBPA was synthesized from adipic acid and diethylenetriamine in a simple one-step process. This malleable covalently crosslinked network polymer can be reshaped and recycled at a high temperature (100 °C) or only with water at ambient temperature (25 °C), which may realize the green processing of energy materials. The introduction of LiTFSI significantly reinforced the conductivity of the dynamic network to a maximum of 3.3 × 10^−4^ S cm^−1^. Another type of PEO-based self-healing solid polymer electrolyte (SHSPE) via dynamically crosslinked imine bonds for safe, flexible and room-temperature LMBs is presented in Figure 6f and Table 1, entry 4 [73]. A PEO-based SHSPE was prepared by the condensation polymerization of poly(ethylene glycol) diamine with 1,3,5-triformylbenzene in dimethylformamide (DMF) solvent. The resulting SHSPE showed an ionic conductivity up to 7.48 × 10^−4^ at 25 °C and excellent mechanical properties with a tensile stress of 137 kPa and a fracture strain of 524%. This material can spontaneously restore its structure and function without extra external stimuli (heat, electricity, etc.) in 24 h at room temperature. The assembled Li|SHSPE|LiFePO_4_ cell demonstrated a brilliant charging/discharging stability at room temperature, with a specific capacity exceeding 126.4 mAh g^−1^ after 300 cycles. A pouch cell was also constructed using the SHSPE, featuring great flexibility and safety, making this material suitable for applications in flexible/wearable electronics. 

In 2022, an interesting study was presented by Deng et al., which is schematically presented in Figure 6g and summarized in Table 1, entry 5 [74]. A self-healing polymer electrolyte PBPE with polymer networks crosslinked by highly reversible imine bonds was fabricated by a Schiff base reaction between poly(ethylene glycol) diamine and benzene-1,3,5-tricarbaldehyde. The PBDE was able to spontaneously repair the cut damage within 1 h at room temperature, while the healed electrolyte exhibited almost the same electrochemical and mechanical performance as the original sample. The ionic conductivity reported for the PBPE (4.79 × 10^−3^ S cm^−1^ at 30 °C) is one of the highest among the self-healing polymer electrolytes. Furthermore, PBPE can promote the generation of LiF-rich SEI and effectively suppress dendrite growth on Li-metal anodes. The LiFePO_4_ (LFP) cells assembled with PBPE exhibited an excellent rate capability (discharge capacity of 118.2 mAh g^−1^ at a 5C rate) and good cycling performance (capacity retention of 97.8% over 125 cycles). More importantly, the cell containing the healed PBPE exhibited a cycle performance identical to the original PBPE (Figure 6h). 

**Figure 7:**
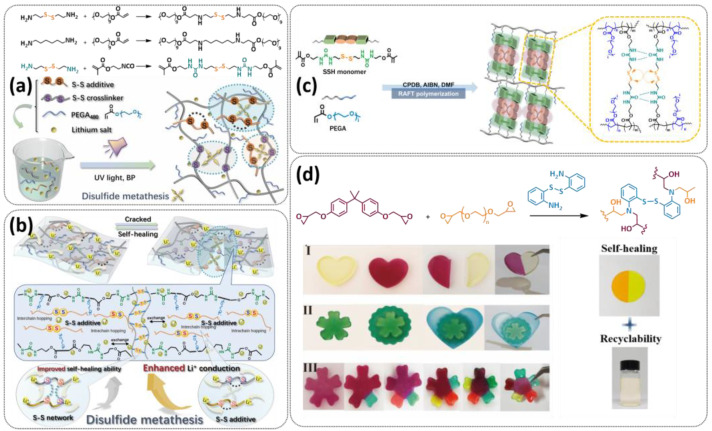
(**a**) Schematic illustration of the fabrication of polymer electrolytes with disulfide bonds (ICEPEs). (**b**) Schematic illustration of lithium-ion conduction, disulfide metathesis and the self-healing process. Reproduced with permission from [84]. Copyright © 2022, American Chemical Society. (**c**) Schematic illustration of the formation of PEG-SSH containing disulfide bonds and hydrogen bonds. Reproduced with permission from [75]. Copyright © 2020, American Chemical Society. (**d**) Schematic illustration of the preparation process and design of self-healing and recyclable polymer electrolyte RFSPE-3 and its self-healing behavior. Reproduced with permission from [83]. Copyright © 2021 Elsevier Ltd. All rights reserved.

In addition, the self-healing mechanism based on disulfide metathesis as the dynamical exchange strategy as investigated for SPEs. Recently, a remarkable approach by combining a disulfide-containing additive with a disulfide-containing polymer matrix to construct polymer electrolytes with enhanced ionic conductivity (ICEPEs) is presented (Figure 7a,b; Table 1, entry 17) [84]. Firstly, the oligo(ethylene oxide)-based additive containing a disulfide bond (S–S additive) was synthesized via a Michael addition reaction of cystamine and poly(ethylene glycol) methyl ether acrylate (PEGA). Subsequently, the precursor fluid was prepared by mixing the S–S additive, PEGA, benzophenone, lithium salt, and crosslinker consisting of a disulfide bond and a urea group (S–S crosslinker), followed by ultraviolet (UV) light polymerization to construct ICEPEs. Short PEG chains complexed with Li^+^ in an S–S additive were able to rapidly migrate in the polymer matrix because of the dynamics of the exchange of the disulfide bond. Moreover, the disulfide bonds in the S–S additive possessed the ability to exchange with the crosslinked network containing disulfide bonds (S–S net). As it can be seen in Figure 7a the self-healing performance of the material (2 h at 80 °C) can be attributed to numerous dynamic disulfide and hydrogen bonds. The prepared ICEPE exhibited an ionic conductivity of 1.24 × 10^−4^ S cm^−1^ at RT, while the assembled Li/ICEPE/LiFePO_4_ cell showed a discharge capacity of 119.9 and 82.5 mAh g^−1^ at 0.2 and 0.3C, respectively, representing capacity retentions of 86.9% and 59.8%. The combination of the urea hydrogen bonds and dynamic disulfide bonds as a self-healing mechanism for SPEs was actually firstly introduced by Jo et al. in 2020 [75]. The self-healing electrolyte PEG-SSH was fabricated by employing reversible addition–fragmentation chain transfer (RAFT) polymerization of poly(ethylene glycol) methyl ether acrylate (PEGA, *M*_n_ = 480) and a crosslinker consisting of hydrogen bond and disulfide bond interactions (see Figure 7c; Table 1, entry 7). The optimized PEG-SSH could heal at room temperature within 30 min, without any external stimuli. Although the PEG-SSH exhibited a moderate ion conductivity of 1.78 × 10^−4^ at 80 °C, the discharge capacity of the Li|PEG-SSH|LFP battery maintained at 131.6 mAh g^−1^ after 100 cycles, representing high-capacity retention (97.5%). More importantly, the self-healed PEG-SSH exhibited a stable cycling performance and could maintain an initial discharge capacity of 128.5 mAh g^−1^. 

Sun et al. fabricated a highly conductive self-healing SPE by introducing disulfide dynamic covalent bonds into an epoxy matrix (Figure 7d; Table 1,s entry 16) [83]. A self-healable and recyclable electrolyte, RFSPE-3, was designed using diglycidyl ether of bisphenol A (DGBE) and poly (ethylene glycol) diglycidyl ether (PEGDGE) for the preparation of the matrix, while 2-aminophenyl disulfide (2-AFD) with disulfide bonds was used as a crosslinker. The following considerations were followed: (1) the coupling of rigid DGEBA and soft PEGDGE is particularly suitable to optimize the trade-off between mechanical properties and ionic conductivity; (2) the dynamic exchange of disulfide bonds in selected 2-AFD endows the electrolyte with an excellent self-healing ability and recyclability. The epoxy resin imparts superior mechanical abilities (tensile strength > 20 MPa) to the electrolytes, while the disulfide bonds provide enhanced self-healing capabilities (healing efficiency > 95%, 2 h at RT). The ion conductivity of RFSPE-3 reached 10^−3^ S cm^−1^, and the multiply healed RFSPE exhibited no changes in the ionic conductivity. In addition, RFSPE-3 showed an inhibitory effect on the growth of lithium dendrites, which resulted in a cycling stability for up to 1800 h at 1 mAh/cm^2^. A somewhat similar approach for the incorporation of disulfide dynamic networks into epoxy matrix was followed by Sun and Wu (see Table 1, entry 15) [82]. The disulfide bond was introduced using a disulfide-containing aliphatic polyamine as the epoxy-curing agent. The prepared SPEs with different LiTFSI contents were optically transparent and self-healable at temperatures above the glass transition temperature (*T_g_*). The curing time to reach the gel point decreased from 102 to 1.4 min by increasing the temperature from 40 to 100 °C. The SPE containing 15% LITFSI exhibited the best ion conductivity of 3.35 × 10^−6^ S cm^−1^ at 80 °C and 8.31 × 10^−6^ S cm^−1^ at 100 °C, which is somewhat lower compared to other self-healing SPEs. 

**Figure 8:**
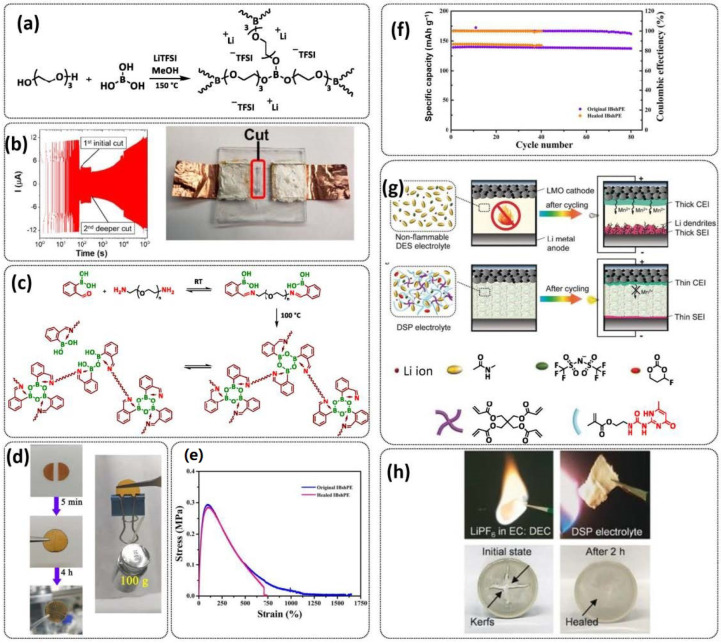
(**a**) Synthesis of dynamic network electrolytes from triethylene glycol and boric acid. (**b**) Current passed at ± 60 °C before and after the network was cut with a razor blade. After 34 h, 98% of the initial current was recovered. Reproduced with permission from [88]. Copyright © 2019, American Chemical Society. (**c**) Synthesis route of IBshPE and self-healing mechanism of a synergetic dynamic imine bond and boroxine bond. (**d**) Self-healing process of IBshPE in the glovebox at RT. (**e**) Stress–strain curves of original IBshPE and healed IBshPE. (**f**) Cycle performance of LFP/original IBshPE/Li cell and of LFP/healed IBshPE/Li cell at a 1C rate. Reproduced with permission from [87]. Copyright © 2022, Elsevier B.V. All rights reserved. (**g**) Deep eutectic solvent (DES) and DES-based self-healing polymer (DSP) electrolytes in Li||LMO cells. (**h**) Optical images of the 1 m LiPF_6_ in EC/DEC electrolyte (top left) and DSP electrolyte (top right) under a combustion test and the self-healing process of the DSP electrolyte after being cut (bottom). Reproduced with permission from [91]. Copyright © 2020, Wiley-VCH Verlag GmbH & Co. KGaA, Weinheim.

B-O dynamic covalent bonds have been extensively studied in different types of self-healing polymers and hydrogels [38]. In 2019, Jing and Evans presented a self-healing SPE based on poly(ethylene oxide) dynamically covalently crosslinked via boric acid and containing different amounts of LiTFSI salt (Figure 8a; Table 1, entry 21) [88]. Their investigations showed that both the ion conductivity (maximum of 3.5 × 10^−4^ S cm^−1^ at 90 °C was reached for optimized composition with Li/EO ratio of 0.085) and mechanical properties (with shear modulus ranging from 1 to 10 MPa) depends strongly on the content of LiTFSI salt. The self-healing ability of the electrolyte was tested by applying an alternating voltage of ±2 V and monitoring current over time (Figure 8b). The current variated around ±12 µA and then dropped to ±3 µA after cutting the network with a razor blade. The SPE fully recovered within approx. 34 h, without any external force. By applying an additional force (19.6 N), self-healing was reached within 10 h. Additionally, these dynamic networks could dissolve in water, yielding a starting monomer, thus presenting a sustainable recyclable alternative. Recently, Zhou et al. presented a self-healing polymer electrolyte (IBshPE) with high ionic conductivity based on a synergetic dynamic imine bond and boroxine bond (Figure 8c; Table 1, entry 20) [87]. IBshPE was prepared via the reaction of 2-formylphenylboronic acid and poly(ethylene glycol) diamine, whereby boroxine bonds with B-N coordination and imine bonds can synchronously undergo fast bond exchange reactions, enabling the rapid self-healing of IBshPE. IBshPE exhibited the highest ionic conductivity (5.08 × 10^−3^ S cm^−1^ at 30 °C) among the reported self-healing polymer electrolytes, while a self-healing efficiency of 97% within 4 h at room temperature was reached (Figure 8d,e). IBshPE facilitates the construction of a robust LiF-rich SEI, thus efficiently inhibiting dendrite growth. LiFePO_4_/Li cells with IBshPE exhibited an excellent cycle performance (capacity retention of 98.6% after 80 cycles) and good rate capability (specific capacity of 130.5 mAh g^−1^ at a 2C rate). More importantly, IBshPE is capable of self-repairing damage in the LiFePO_4_/Li cells and restoring the performance of the cells (Figure 8f).

The last example in this section can actually be regarded as being on the borderline between SPEs and GPEs. A stable quasi-solid-state Li-metal battery with a deep eutectic solvent (DES)-based self-healing polymer (DSP) electrolyte was reported in 2020 by Jaumaux et al. [91]. The DSP electrolyte was synthesized in situ by thermally polymerizing 2-(3-(6-methyl-4-oxo-1,4-dihydropyrimidin-2-yl)ureido)ethyl methacrylate (UPyMA) and pentaerythritol tetraacrylate (PETEA) monomers in the presence of a DES-based electrolyte, which contained LiTFSI and *N*-methylacetamide (NMA) in an eutectic ratio, and fluoroethylene carbonate (FEC) as an additive (Figure 8g; Table 1, entry 24). The addition of FEC significantly improved the ionic conductivity and electrochemical stability of the DSP electrolyte. It simultaneously constructed a robust fluorine-rich SEI and a cathode electrolyte interface (CEI) against Li dendrite growth on the anode and structural deterioration of the cathode, respectively. The self-healing UPyMA–PETEA copolymer matrix kept the DSP electrolyte in a quasi-solid-state form without the safety issues of electrolyte leakage and maintained excellent electrode|electrolyte interfacial contacts during prolonged cycling. The as-developed DSP electrolyte exhibited a self-healing ability (H-bonds, 2 h at RT), noncombustibility (Figure 8h), high ionic conductivity (1.79 × 10^−3^ S cm^−1^ at 25 °C) and electrochemical stability (up to 4.5 V vs. Li/Li^+^), as well as stable interfacial properties. When applied in Li-metal||LiMn_2_O_4_ (LMO) batteries, the DSP electrolyte effectively suppressed manganese dissolution from the cathode and enabled a high capacity and a long lifespan at room and elevated temperatures. 

### 3.3. Self-Healing Gel Polymer Electrolytes (GPEs)

GPEs, in general, consist of a liquid component embedded in a polymer matrix (either physically mixed or chemically crosslinked), thus combining the mechanical integrity of polymeric materials with the enhanced ion conductivity of liquids. GPEs have been developed as an intermediate between liquid electrolytes and SPEs in order to improve the limited ion conductivity of SPEs [120,121]. They mostly consist of a polymer matrix, lithium salts, solvent (plasticizer) and potential further additives. The self-healing functionality and mechanical integrity is provided by self-healing polymers, which also minimize safety issues by preventing the leaching of the liquid components. Li-ions dissolved in plasticizer govern ion transport. The most investigated plasticizer for self-healing GPEs are ionic liquids [115,122], but other types of solvents can also be used, such as ethylene carbonate (EC), diethyl carbonate (DEC), 1,2-dioxolane (DOL) or dimethoxymethane (DME). In general, the design of self-healing GPEs presents a balancing act in order to find an optimal polymer matrix/plasticizer ratio/interaction. On the one hand, GPEs exhibit significant advantages compared to liquid electrolytes, such as excellent electrode/electrolyte interface properties, good mechanical integrity and enhanced safety [35,120,121]. On the other hand, although the higher liquid content in GPEs results in high ion conductivity (10^−4^–10^−3^ S cm^−1^), simultaneously a higher liquid content negatively influences the mechanical and thermal stability of a gel. Thus, the choice of type and amount of plasticizer is of paramount importance when designing GPEs. 

**Figure 9:**
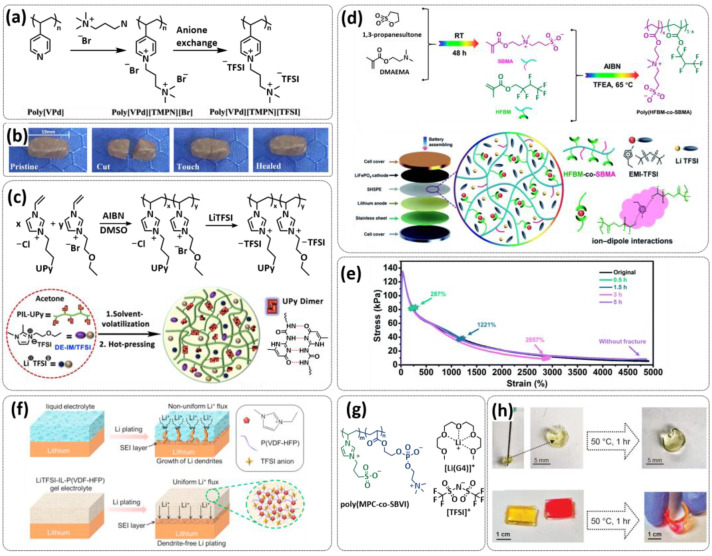
(**a**) Synthesis route towards self-healing PVT polymeric ionic liquid. (**b**) Photographs of the PVT-40%EMITFSI electrolytes during the self-healing process. Reproduced with permission from [96]. Copyright © 2019, Elsevier B.V. All rights reserved. (**c**) Schematic illustration of the synthesis of the PIL-UPy copolymers and the preparative process of the ionogel membranes. Reproduced with permission from [95]. Copyright © 2019, American Chemical Society. (**d**) Synthetic route towards poly(HFBM-*co*-SBMA), schematic illustration of the configuration of the Li/poly(HFBM-*co*-SBMA) ionogel/LiFePO_4_ cell and self-healing mechanism of poly(HFBM-*co*-SBMA) ionogel. (**e**) Tensile curves of poly(HFBM-*co*-SBMA) ionogel at various self-healing times at room temperature. Reproduced from Reference [93] with permission from the Royal Society of Chemistry. (**f**) Schematic illustration of the electrochemical deposition behavior of lithium metal anodes with: liquid organic solution electrolyte (above) and LiTFSI-IL-P(VDF-HFP) gel electrolyte (below). Reproduced with permission from [92]. Copyright © 2018, Elsevier B.V. All rights reserved. (**g**) Molecular structure of (Li(G4))^+^, TFSI^−^ anion and fully zwitterionic copolymer scaffold consisting of MPC and SBVI monomers. (**h**) Self-healing behavior of zwitterionic ionogel. Reproduced with permission from [97] Copyright © 2019, American Chemical Society.

We already discussed that ILs present an auspicious alternative for lithium batteries due to the fact of their unique properties, such as high electrochemical and thermal stability, ion conductivity and fire retardancy. Thus, it is not surprising that ILs are the most investigated plasticizers in GPEs (so called ionogels) [8,115,122]. In 2020, Tian et al. presented a stretchable, self-healing GPE based on the self-healing dicationic polymeric liquid (see Figure 9a; Table 1, entry 29) as a backbone filled with ionic liquid (EMIMTFSI) within interspaces among chain segments [96]. In the optimized GPE (PVT-40%EMITFSI), 40 wt% IL-plasticizer was used. Self-healing was achieved via ionic interactions, and the sample was fully healed after 60 min at RT (Figure 9b). Both a pristine electrolyte block and the healed one could be stretched by over 700%, indicating a good flexibility performance. The ambient temperature conductivity of PVT-45%EMIMTFSI was up to 1.26 × 10^−4^ S cm^−1^, much higher than that of PVT-40%EMIMTFSI (3.55 × 10^−5^ S cm^−1^), indicating that the IL content governs the conductivity of the PVT-EMIMTFSI electrolytes. The Li/PVT-45%EMIMTFSI/LiFePO_4_ battery showed an initial discharge capacity of 145 mAh g^−1^. The working group of Sun successfully combined quadrupole hydrogen bonding of the UPy group with ionic interaction of PILs/ILs to fabricate self-healing GPEs [95]. As it can be seen in Figure 9c (Table 1, entry 28), they firstly prepared imidazolium-based random copolymer via free radical polymerization by using UPy- and ethoxyethyl-functionalized imidazolium-IL monomers (molar ratio IL-UPy:IL-ether = 2:1). As a plasticizer, an IL 1,2-dimethyl-3-ethoxyethyl imidazolium bis(trifluoromethanesulfonyl)imide (labeled as DE-IM/TFSI) was used and LITFSI was added as Li-salt. The ionogel electrolytes exhibited ionic conductivities as high as 10^−3^ S/cm, which is comparable to the conventional liquid electrolytes. The Li/LiFePO_4_ battery assembled with the ionogel membrane exhibited an excellent cycling performance and delivered a steady high discharge capacity of 147.5 mA h g^−1^ and a Coulombic efficiency of 99.7% after 120 cycles at the charge/discharge rate of 0.2C. The healed ionogel, after being stored at 55 °C for 1 h, presented 70.5% and 79.3% recoveries of tensile strength and stress. 

Wang et al. fabricated a highly stretchable, nonflammable and notch-insensitive intrinsic self-healing ionogel [93]. They prepared a zwitterionic copolymeric ionic liquid with fluorinated side chains (poly(HFBM-*co*-SBMA) by the radical copolymerization of 2,2,3,4,4,4-hexafluorobutyl methacrylate (HFBM) and sulfobetaine methacrylate (SBMA) (Figure 9d; Table 1, entry 26). For the preparation of ionogels, ionic liquid EMIMTFSI and LITFSI salt were added. The underlying mechanism is based on ionic interaction and ion–dipole interactions between fluorinated chains and imidazolium cations of the IL. The damaged ionogel was able to repair under ambient conditions within 60 min. Due to the presence of ionic liquid within the zwitterionic polymer matrix, the obtained ionogel exhibited remarkable stretchability (elongation of 4000% without fracture) and could recover mechanical properties after 5 h (Figure 9e). It should be mentioned that already in 2018, Chen and coworkers presented a self-healing ionogel electrolyte LiTFSI-IL-P(VDF-HFP) constructed by soaking a P(VDF-HFP) (poly(vinylidene fluoride)-co-hexafluoropropylene) polymer matrix in ionic liquid EMIMTFSI (40% wt.) and 1 M LiTFSI (Figure 9f; Table 1, entry 25). Strong and robust ion–dipole interactions between fluorine-containing P(VDF-HFP) and imidazolium cations resulted in ionogels with good self-healing ability (12 h at RT), good ion conductivity (8.8 × 10^−4^ S cm^−1^ at room temperature), high Young’s modulus of 6.8 GPa (four times higher than that of pristine P(VDF-HFP)) and flame retardancy. 

D’Angelo and Panzer, for the first time, presented self-healing ionogels based on fully zwitterionic polymer networks [97]. Fully zwitterionic (f-ZI) polymer scaffold-supported solvate ionogels were synthesized via the UV-initiated free-radical (co)polymerization of two zwitterionic monomers, 2-methacryloyloxyethyl phosphorylcholine (MPC) and sulfobetaine vinylimidazole (SBVI), in situ within the solvate ionic liquid (Li(G4))(TFSI), which was prepared from an equimolar mixture of lithium bis(trifluoromethylsulfonyl)imide (LiTFSI) and tetraglyme (G4) (Figure 9g; Table 1, entry 30). The mechanical properties of ionogels can be varied by the ratio of MPC and SBVI monomers in poly(MPC-co-SBVI). The ionomers with a higher SBVI fraction were stiff and brittle, whereas the MPC-dominated ionogels were soft and stretchable. Thus, due to the fact of their higher chain mobility, the MPC-dominated ionogels exhibited self-healing properties via ionic and dipole interactions, as well as due to the fact of molecular diffusion. The efficient self-healing of ionogel was achieved after 1 h at 50 °C (Figure 9h). 

**Figure 10:**
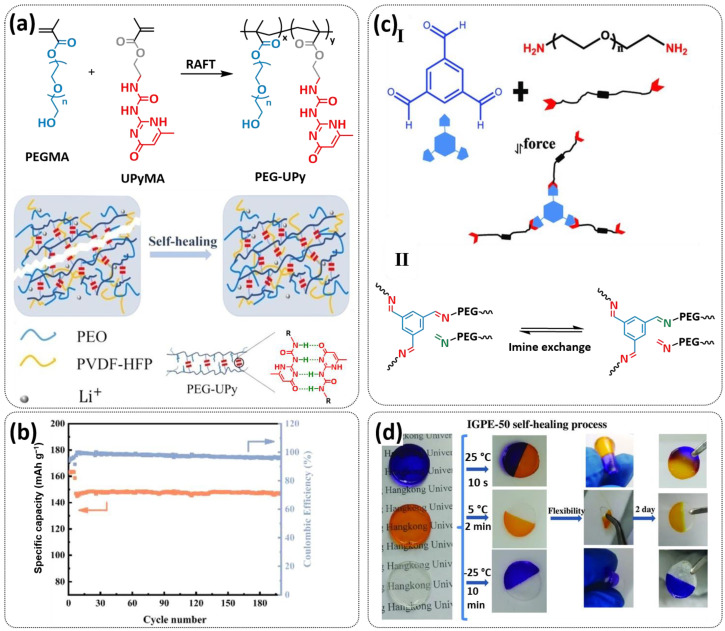
(**a**) Schematic illustration of the synthesis of brush-like copolymer PEG-UPy and the formation of the self-healing GPE. (**b**) Cycling performance and Coulombic efficiency of the Li/GPE-20/LFP cell at 1C. Reproduced with permission from [100]. Copyright © 2022, American Chemical Society. (**c**) Schematic representation of the reversible dynamic imine bond in the IGPE. (**d**) Self-healing process of IGPE-50 at different temperatures. Reproduced with permission from [99]. Copyright © 2021, Wiley-VCH GmbH.

An interesting example of a self-healing GPE without IL-plasticizer was presented by Wang’s research group [100]. The GPE was based on the crosslinking network formed by adding supramolecular copolymer PEG-UPy to the crosslinking matrix of poly(ethylene oxide) (PEO) and polyvinylidene fluoride hexafluoropropylene (PVDF-HFP) (Figure 10a; Table 1, entry 33). A brush-like copolymer (PEG-UPy) was synthesized by the RAFT polymerization method using PEG containing methacrylate monomer (PEGMA) and methacrylate monomer containing a UPy group (UPyMA). The GPE film was prepared by mixing the PEGMA-UPyMA copolymer, PEO and PVDF-HFP in distinct ratios and applying the coating method. The optimized electrolyte, GPE-20, had a good ionic conductivity of 7.5 × 10^−4^ S cm^−1^ at 25 °C. The GPE-20 exhibited a good self-healing capability at 25 °C (15 h) and good interfacial stability with the electrode. The discharge-specific capacity of the Li/GPE-20/LFP cell reached 161.5 mAh g^−1^ at 0.05C and kept 132.6 mAh g^−1^ at 1C. Then, the current rate returned to 0.05C, and the capacity could recover to 159.3 mAh g^−1^ (Figure 10b).

Over the last couple of years, impactful research towards the implementation of self-healing materials based on dynamic covalent bonds into GPEs has been conducted. Wan et al. developed a chemically crosslinked ionic gel polymer electrolyte (IGPE) with good self-healing ability generated by dynamic imine covalent bonds [99]. The IGPE was designed in a polycondensation reaction of polyoxyethylenebis(amine) (NH_2_–PEG–NH_2,_ M_n_ = 2000 Da) and 1,3,5-benzenetricarboxaldehyde (TBP) in the presence of ionic liquid BMImTFSI—1-butyl-3-methylimidazolium bis(trifluoromethanesulfonul)imine (0.1 mol kg^−1^ LiTFSI/BMImTFSI) through a one-step reaction (Figure 10c; Table 1, entry 32). The interaction between IL and the ether oxygen of the PEG chains improved the stability of the IGPE, and the crystallinity decreased significantly due to the incorporation of ionic liquid. The IGPEs showed good self-healing (10 s at 25 °C, Figure 10d), film flexibility and high ionic conductivity even at −25 °C (7.2 × 10^−4^ S cm^−1^ at ambient conditions and 1.6 × 10^−5^ S cm^−1^ at −25 °C). They also possessed a high thermal stability, wide electrochemical stability window and good interfacial compatibility. The Li/IGPE-50/LiFeO_4_ battery showed a high discharge capacity of 154.8 mAh g^−1^ at 0.1C with 99.6% Coulombic efficiency, while remaining at 132.8 mAh g^−1^ at the 50th cycle. 

**Figure 11:**
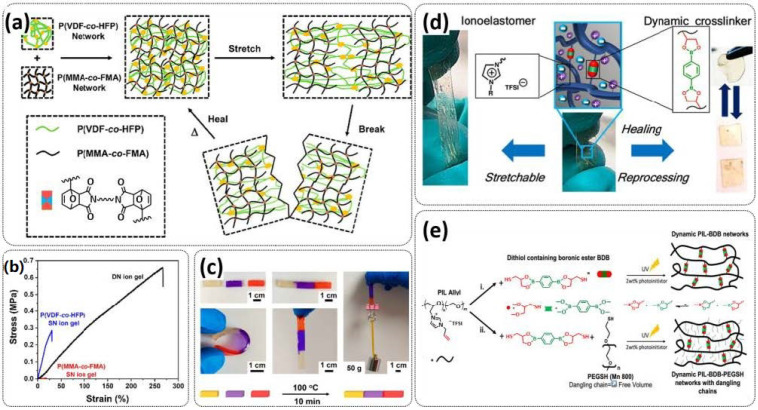
(**a**) P(FMA-*co*-MMA)/P(VDF-*co*-HFP)/(EMIM)(TFSI) DN ion gel and its self-healing mechanism. (**b**) Tensile stress–strain curves of the DN and the respective SN ion gels. (**c**) Thermal healing capability of the DN ion gel. Reproduced with permission from [94]. Copyright © 2018, American Chemical Society. (**d**) Concept and (**e**) synthetic approach towards a vitrimeric ionoelastomer. Reproduced from [98].

Tang et al. fabricated a tough double-network (DN) ion gel composed of chemically crosslinked poly(furfuryl methacrylate-*co*-methyl methacrylate) (P(FMA-*co*-MMA)) and physically crosslinked poly(vinylidene fluoride-*co*-hexafluoropropylene) (P(VDF-*co*-HFP)) networks with 80 wt% ionic liquid (EMIMTFSI) via a one-pot method (Figure 11a; Table 1, entry 27) [94]. Thereby, (P(FMA-*co*-MMA)) is a brittle chemically crosslinked network, while (P(VDF-*co*-HFP)) is a ductile physically crosslinked network. For crosslinking, the thermally reversible Diels–Alder (DA) reaction between furan moieties on the side chain and a multi-maleimide crosslinker to construct the chemically crosslinked network was utilized. Upon an adjustment of the weight ratio of P(FMA-*co*-MMA) to P(VDF-*co*-HFP) and the content of the crosslinker, a remarkably robust DN ion gel (failure tensile stress: 660 kPa, strain: 268%; failure compressive stress: 17 MPa, strain: 85%) was obtained (Figure 11b). The high mechanical strength is attributed to the chemical/physical interpenetrating networks. The cracked film sample healed itself in only 10 s at 100 °C (Figure 11c). 

Most recently, Plesse and coworkers reported a healable ionoelastomer designed by combining supramolecular ionic interactions of polymeric ionic liquid with the dynamic vitrimer chemistry of borate ester bonds (Figure 11d; Table 1, entry 31). Firstly, polymeric ionic liquid bearing an allyl functional group (PIL allyl) was synthesized by the quaternization of *N*-allylimidazole with a high molecular weight and commercially available poly(epichlorohydrin-*co*-ethylene oxide) rubber (Figure 11e). The ionoelastomer was then obtained by the crosslinking of PIL allyl with di-thiol crosslinkers through the thiol–ene click reaction. The presence of the dynamic boronic ester function in the crosslinker structure turned the ionoelastomer into a reprocessable and healable vitrimer. The obtained ionoelastomer could be healed after cutting within 2 h at 120 °C. As expected, the ionic conductivity of ionoelastomer depends strongly on the exact composition (PIL/vitrimer ratio) and degree of crosslinking. For the optimized ionoelastomer, values up to 1.6 × 10^−5^ S cm^−1^ at 30 °C were obtained. The conducted recycling tests reveal that the dynamic exchange reaction of the boronic ester bonds allowed for the topological rearrangement of the covalently crosslinked ionoelastomer networks, enabling them to be reshaped and recycled. However, a detail electrochemical investigation of ionoelastomers to estimate their applicability in lithium batteries is still pending. 

### 3.4. Self-Healing Composite Polymer Electrolytes (CPEs)

In general, composite polymer electrolytes (CPEs) are prepared by mixing inorganic fillers with polymer electrolytes. The role of inorganic fillers is to enhance the mechanical features of the polymer matrix while simultaneously increasing the ionic conductivity. There are two types of fillers: nonion conductive and ion conductive fillers [43,123,124]. Additionally, it is possible to differentiate between a composite gel-state and composite solid-state electrolyte; however, we did not make this distinction within this paper but rather highlighted the general progress in the research field of CPEs. 

**Figure 12:**
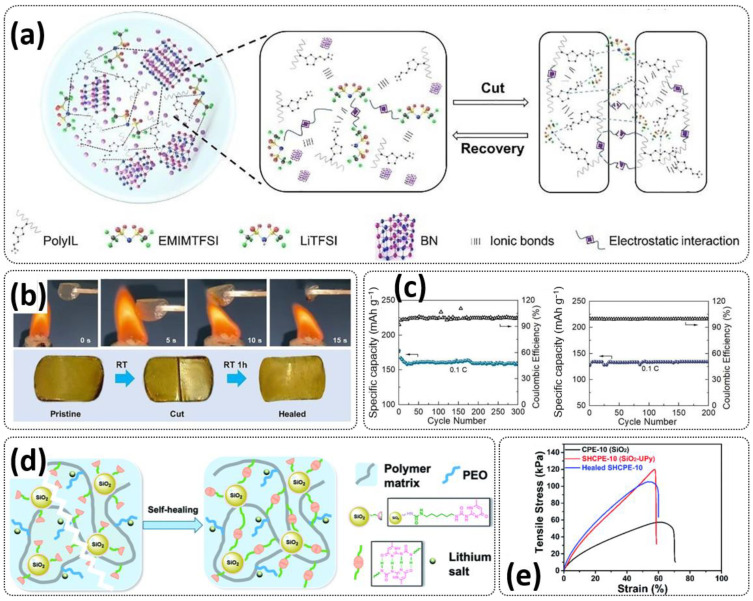
(**a**) Illustration of the self-healing mechanism of PolyIL-5. (**b**) Burning test and self-healing of PolyIL-5. (**c**) Long-time cycling performance of the LFP/Li cell with PolyIL-5 electrolyte before and after healing. Reproduced with permission from [106]. Copyright © 2022 The Authors. Published by Elsevier B.V. (**d**) Schematic illustration of the structure of the SHCPE with the supramolecular networks. (**e**) Mechanical properties of the CPE-10, SHCPE-10 and healed SHCPE-10 samples. Reproduced from Reference [102] with permission from the Royal Society of Chemistry.

A flexible, self-healing composite solid electrolyte (CSE) composed of PolyIL, 2D boron nitride (BN) nanosheets, 1-ethyl-3-methylimidazoliumbis (trifluoromethylsulfonyl)imide (EMIMTFSI) and bistrifluoromethanesulfonimide lithium salt (LiTFSI) was constructed via a template route (Figure 12a; Table 1, entry 38) [106]. In this work, PolyIL (poly(N,N,N-trimethyl-N-(1-vinlyimidazolium-3-ethyl)-ammonium bis(trifluoromethanesulfonyl)imide) (poly-(VIm)(TMEN)(TFSI))) was selected because of its relatively low operation temperature and high ionic conductivity. EMIMTFSI, which is highly ionic conductive with low viscosity, was used to decrease the interface resistance [125]. Boron nitride (BN) nanosheets with a high specific surface area and richly porous structure were used as the passive inorganic filler, where insulating the electron feature of BN avoids short circuits, and the uniform and wide channels between and outside the BN layers form a pathway for the Li^+^ transport, homogenizing the Li^+^ flux and facilitating the Li^+^ transmission. The optimized electrolyte, PolyIL-5, exhibited an enhanced conductivity of 0.158 × 10^−3^ S cm^−1^ at 25 °C and 1.487 × 10^−3^ S cm^−1^ at 85 °C, while the lithium transference number t_Li_^+^ = 0.53 could be reached. The electrolyte is flexible, nonflammable and could recover after cutting to the initial state within 1 h a room temperature (Figure 12b). The assembled LFP/PolyIL-5/Li cell retained 132.4 mAh g^−1^ and 87% specific capacitance at 0.1C for 200 cycles after healing (Figure 12c). 

Zhou et al. presented a supramolecular network-reinforced self-healing composite solid-state polymer electrolyte (SHCPE) using a UPy functionalized silica filler and UPy functionalized PEG-based polymer matrix to form a crosslinked hydrogen bonding structure (Figure 12d; Table 1, entry 35) [102]. The SiO_2_-UPy fillers enhanced the active crosslinking in the polymer matrix, thus leading to the self-healing and mechanical features of the material. The SHCPE-10 with 10% wt. SiO_2_-UPy was subjected to a tensile stress of 120 kPa, which is significantly higher than the value of PEG-UPy (30 kPa), indicating that the UPy groups on the SiO_2_-UPy’s surface actively enhanced the crosslinking of the polymer matrix (Figure 12e). The CPE SHCPE-10 showed an ion conductivity of 8.01 × 10^−5^ S cm^−1^ and could be fully repaired after 60 min at RT. In addition, the battery assembled with SHCPE-10 showed long-term cycling performance. The discharge capacity was maintained at 139 mA h g^−1^ with a Coulombic efficiency of 97.9% after 60 cycles. 

**Figure 13:**
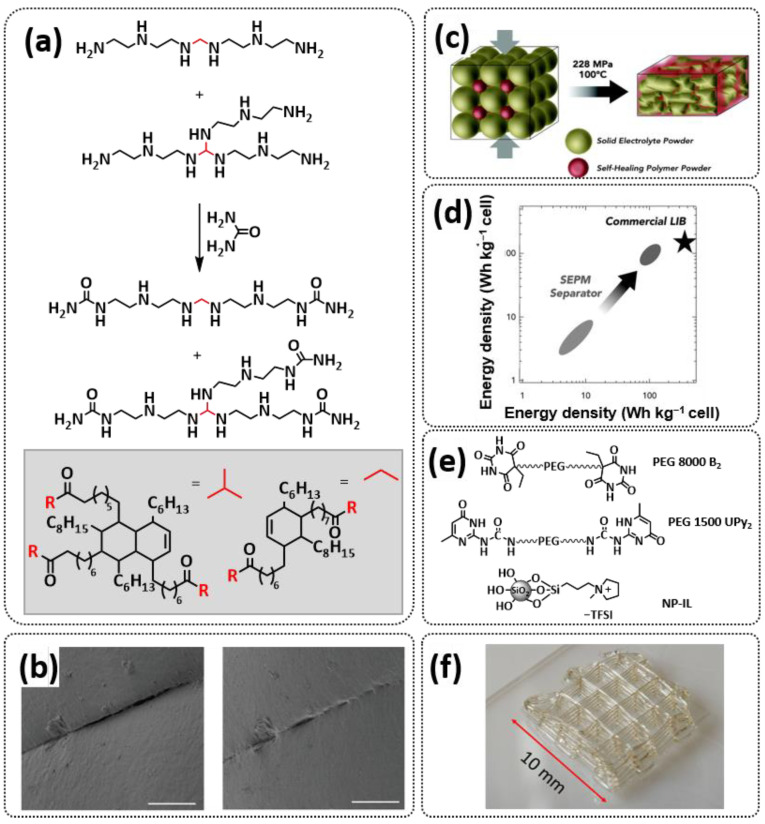
(**a**) Synthesis route of fatty acid-based SHP. (**b**) SEM images of the CPE surface showing the self-healing functionality after 1 h. Scale bar: 100 µm. Reproduced from Reference [101]. (**c**) Schematic for forming the solid electrolyte in polymer matrix membrane (SEPM). (**d**) Enhancement of the gravimetric and volumetric energy densities by moving to an SEPM configuration. Reproduced with permission from [103]. Copyright © 2015 WILEY-VCH Verlag GmbH & Co. KGaA, Weinheim. (**e**) Chemical structures of the materials used for the preparation of 3D-printable CPEs. (**f**) Printed CPE. Reproduced from Reference [105].

Xia et al. reported an LLZGO-based (garnet Ga-doped Li_7_La_3_Zr_2_O_12_) composite polymer electrolyte combined with a fatty-acid-based self-healing polymer matrix (SHP) [101]. Fatty acids are used as monomers to provide self-healing features via successive amidation and urea treatment (Figure 13a; Table 1, entry 34). The self-healing composite polymer electrolyte membrane was fabricated by infiltration of the SHP/LLZGO composite with an LiPF_6_ liquid electrolyte by the slurry coating method. Due to the multiple hydrogen bonds in the SHP, the membrane could be repaired in 1 h under ambient conditions (Figure 13b). The prepared composite electrolyte displayed strong self-adherence to lithium metal, which efficiently reduces the interfacial incompatibility, thus inducing a stable SEI layer to inhibit Li-dendrite formation. Whiteley et al. designed a CPE solid-state film by hot pressing a mixture of Li_2_S-P_2_S_5_ inorganic electrolyte and self-healing polyimine (SEPM, Figure 13c; Table 1, entry 36) [103]. The reversible imine covalent bonds enabled the formation of a continuous crosslinked polymer network between the solid electrolyte gaps, thus increasing the mechanical integrity of the SEPM without a large loss of ion conductivity. The prepared SEPM could reach up to an 80% solid electrolyte load and ion conductivity up to 5.2 × 10^−4^ S cm^−1^. The SEPM, when used as a separator in an all-solid-state battery with an FeS_2_-based cathode, achieved an excellent rate capability and stable cycling for over 200 cycles. By replacing the thick a77.5 separator with the methyl-imine SEPM, the cell-level energy density values increased by an order of magnitude (SEPM, Figure 13d). 

Recently, we designed 3D printable composite polymer electrolytes based on a modified PEG-polymer matrix containing either ureidopyrimidone (UPy) or barbiturate hydrogen-bonding moieties, lithium bis(trifluoromethylsulfonyl)imide (LiTFSI) and IL-surface-modified SiO_2_-based nanofillers (Figure 13e; Table 1, entry 37) [105]. The composite electrolyte PEG 1500 UPy_2_/LiTFSI (EO:Li 5:1) mixed with 15% NP-IL reached an ion conductivity up to 2.8 × 10^−5^ S/cm at 80 °C and was successfully 3D printed (Figure 13f), revealing its suitability for application as printable composite electrolytes. Further, electrochemical investigations of this interesting class of materials are currently ongoing. In general, the design of printable self-healing polymeric materials for application in batteries may be an interesting approach towards better processability and novel battery architectures [126,127]. 

## 4. Self-Healing Binders for Lithium Batteries 

Although graphite carbon holds the main market of anodic materials in commercial LIBs due to the fact of its cycling stability, the low theoretical capacity (372 mAh g^−1^, LiC_6_) hinders its further development and applications in high-energy LIBs [5]. One of the promising next-generation anode materials for lithium batteries is lithium metal (LM) because of its high theoretical specific capacity (3860 mAh g^−1^) [128,129,130,131,132]. However, the safety issue and the uncontrollable Li dendrite growth are still difficult to overcome [18,133,134]. Another promising anode material for next-generation high-energy density LIBs is the intercalation-type silicon (Si) anode, which offers the highest known theoretical specific capacity, approximately ten times higher than that of conventional graphite anodes (4200 mAh g^−1^, Li_4.4_Si), accommodating over four Li^+^ per Si [132,135,136]. However, its practical application is seriously impeded by its mechanical and electrochemical instability. Thus, the introduction of self-healing polymeric materials in order to increase the electrochemical capacity and durability of electrode materials is of great importance for next-generation lithium batteries.

The progress in self-healing polymer binders for Si- and Li-metal anodes is summarized in Table 2 for comparison and discussed in detail in the following sections.

### 4.1. Self-Healing Binders for Si Anodes

**Figure 14:**
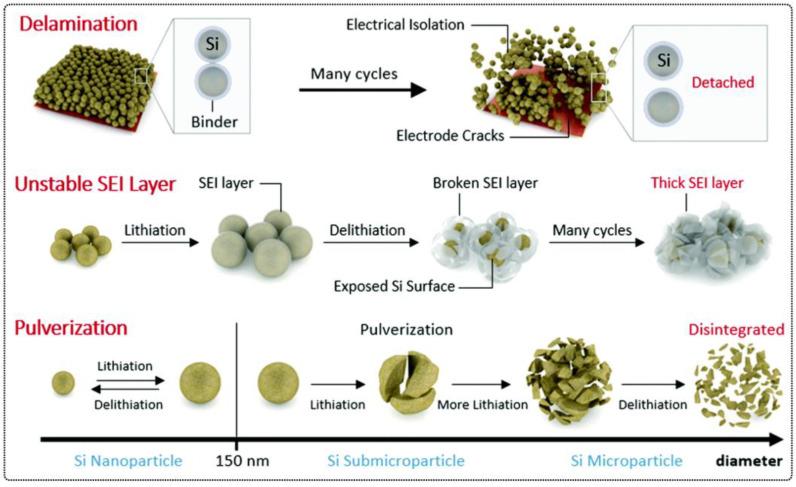
The typical failure mechanisms of Si anodes, including delamination between the different Si particles and current collector, the formation of an unstable SEI layer and the pulverization of Si microparticles. Reproduced from [159] with permission from the Royal Society of Chemistry.

As previously mentioned, the main drawback of Si anodes in lithium batteries is connected to the rapid capacity fading during cycling and poor cycle life. This is mainly because silicon expands volumetrically by up 300% upon full lithium insertion (lithiation) and contracts significantly upon lithium extraction (delithiation). These extreme volumetric changes can cause cracking and pulverization in the electrode, which lead to separation from conductive additives (loss of electrical contact) and excessive solid–electrolyte interphase (SEI) growth [157,158]. The description of the failure mechanism of Si anodes during lithiation/delithiation is presented in Figure 14 [35,159]. Firstly, repeated volumetric changes due to the expansion and shrinkage of Si particles lead to the loss of particle-to-particle contacts, followed by the electrical isolation of Si particles and the delamination between composite electrodes and current collectors. Secondly, parts of the organic electrolytes form a thick and unstable solid electrolyte interphase (SEI) layer on the electrode surface. Repeated volume changes damage the SEI layer, resulting in the formation of an additional SEI layer on the freshly cracked Si surface. Due to the fact of these processes, Li-ions and electrolytes are continuously consumed, thus shortening the lifetime of a battery. In general, two types of Si particles can be applied: micro- and nano-particles One significant advantage of Si nanoparticles (SiNPs) over Si-microsized particles (SiMPs) as anode materials is that they have a stronger mechanical tolerance to endure volume change than SiMPs and, therefore, demonstrate better cycling performance [160,161]. However, the synthesis of SiNPs is cost intensive, and a good dispersion in a matrix is still a challenge. In addition, severe side reactions with electrolyte due to the large specific surface and agglomeration, as a result of electrochemically driven sintering, reduces the electrochemical performance of SiNPs, since the traditional binder is not capable enough to maintain an Si electrode’s integrity upon cycling [162,163]. Thus, the introduction and development of self-healing polymer binders for Si anodes are crucial for their implementation in next-generation lithium batteries. 

**Figure 15:**
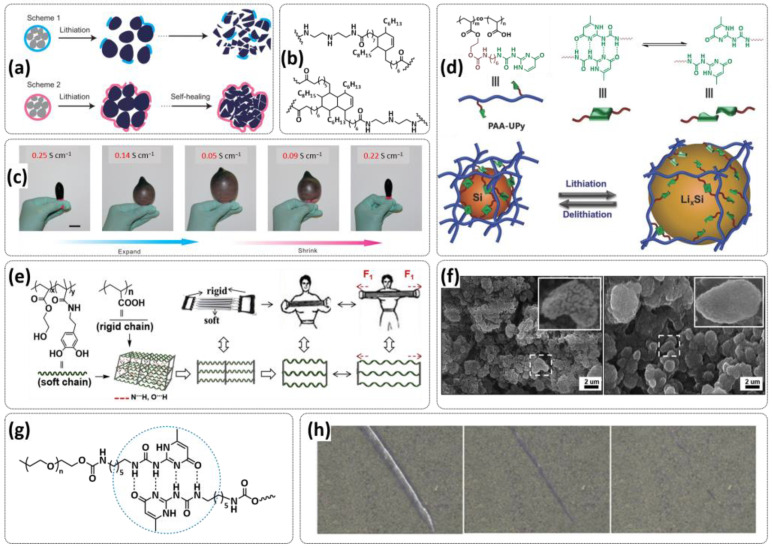
(**a**) Schematic illustration of the self-healing concept of polymer binder for Si anodes. (**b**) Chemical structure of the SHP binder. (**c**) The SHP/CB composite material was coated onto an inflatable balloon to mimic the volumetric changes of silicon particles over the cycling process. The changes in its electrical conductivity during the balloon’s repeated cycles of inflation and deflation were monitored. The SHP/CB coating remained conductive over the whole expand/shrink process. Scale bar: 2 cm. Reproduced with permission from [24]. Copyright © 2013 Nature Publishing Group. (**d**) Schematic illustration of the charge–discharge process of silicon anodes using self-healable PAA–UPy polymer as a binder and the chemical structure of the PAA–UPy supramolecular polymer. The UPy–UPy dimers could reversibly break and reform based on quadruple hydrogen bonding. A large volume expansion of silicon particles during the charge process resulted in the dissociation of the noncovalent crosslinking of UPy dimers, and these crosslinking networks could be rebuilt when the battery underwent delithiation process even after many cycles due to the reversibility of quadruple hydrogen bonding. Reproduced with permission from [141]. Copyright © 2018 WILEY-VCH Verlag GmbH & Co. KGaA, Weinheim. (**e**) Chemical structures and illustrative interaction of P(HEA-co-DMA) and PAA and the spring expanders model of their complex. (**f**) SEM images of SiMP electrodes after 100 cycles for PAA binder (left) and PAA-P(HEA-co-DMA) binder (right). Reproduced with permission from [142] Copyright © 2018 Elsevier Inc. (**g**) Proposed self-healing mechanism of the UPy-PEG binders. (**h**) Self-healing behavior on a Py-PEG-Si-15 electrode. Reproduced with permission from [143]. Copyright © 2017 Elsevier B.V. All rights reserved.

The pioneering work of Bao and coworkers in 2013 [24] inspired worldwide research on self-healing polymer binders for Si anodes. Bao’s research group used a concept previously reported by Leiblers’s group for supramolecular rubbers [164] to develop self-healing polymer (SHP) coatings for Si electrode based on a urea-based hydrogen-bonding self-healing binder (Table 2 entry 1). The concept of the SHP binder and its functionality is presented in Figure 15a,b. Carbon black (CB) is used as a conductive additive. The degree of crosslinking could be variated by reaction conditions and temperature, resulting in polymer networks with a T_g_ of approximately 0 °C, capable of self-healing. In contrast to conventional binders, SHP could compensate for the internal mechanical stress caused by the volume changes of SiMPs while still remaining conductive (Figure 15c) and autonomously repair the microcracks, prolonging the cycling life of the Si anode. By further applying this type of SHB but varying the ratio of hydrogen bonding sites, the same working group demonstrated a high areal capacity (3–4 mAh cm^−2^) and stable cycling for more than 140 cycles using low-cost large Si particles (Table 2 entries 2 and 3) [137,138]. Additionally, they also combined the urea hydrogen bonding SHP with PEG (Table 2, entry 4) [139]. The polymer’s PEG side groups can alleviate the charge transfer resistance between Si particles and electrolytes, as they can facilitate Li-ionic conduction to enable a good rate performance. A previously developed SHP binder was also successfully applied for the construction of a freestanding SiMPs/SHP/CB composite electrode, which does not require a separate current collector (Table 2, entry 4). The electrode demonstrated a 91.8% capacity retention after 100 cycles at C/10 rate, with an average specific capacity and gravimetric capacity, including a current collector mass of ∼2100 mA h g^−1^ and ∼1050 mA h g^−1^, respectively, which is a significant improvement compared to the conventional design of simple self-healing polymer coatings on silicon particle-embedded current collectors [140]. 

Deng and coworker’s presented a quadrupole-bonded supramolecular polymer that can serve as a self-healable binder for high-performance silicon nanoparticle (SiNP) anodes through covalently integrating a small amount of UPy moieties with a linear polymer poly(acrylic acid) (PAA) (Figure 15d; Table 2, entry 6) [141]. PAA itself has been widely investigated as a binder for Si electrodes due to the presence of large number of carboxylic acid groups capable of undergoing hydrogen bonding among each other and with Si anodes, thus providing a good electrochemical stability of the electrode during cycling. The incorporation of UPy groups results in a supramolecular polymer, which offers a strong adhesion strength with SiNP able to withstand large volume expansion and release of the internal stress of SiNP during repeated lithiation/delithiation. The electrodes using this supramolecular binder demonstrated an initial discharge capacity of 4194 mAh g^−1^ and a Columbic efficiency of 86.4%, which represents a significant improvement of the electrochemical properties in comparison to those using PAA, carboxyl methylcellulose (CMC) and poly(vinylidene fluoride) (PVDF) binders. Furthermore, a high capacity of 2638 mAh g^−1^ was achieved after 110 cycles. Another type of self-healing PAA-based binder is presented by Xu et al. [142]. They designed water soluble poly(acrylic acid)-poly(2-hydroxyethyl acrylate-co-dopamine methacrylate) PAA-P(HEA-co-DMA) binder for SiMPs (Table 2 entry 7). PAA-P(HEA-co-DMA) binder is prepared through the in situ thermal condensation reaction of PAA with poly(2-hydroxyethyl acrylate-co-dopamine methacrylate) (P(HEA-co-DMA) in a weight ratio of 4:1. The design concept includes the formation of a self-healing double-network structure; the covalently crosslinked structure forms a scaffold while abundant hydrogen bonds exist in every local area (Figure 15e). A rigid PAA component of the crosslinked network increases the binding force with Si particles by H-bonds between carboxylic acid groups and Si particles, while the soft, viscoelastic P(HEA-co-DMA) fraction prevents flow and creep to compensate for volume changes. A coated Si electrode exhibited good cycling stability within 200 cycles at 0.4 Ag^−1^. A postmortem investigation by scanning electron microscopy (SEM) revealed that the SiMP electrode using PAA-P(HEA-co-DMA) binder maintained a smooth surface morphology and intact particles even after 100 cycles (Figure 15f, right), whereas the electrode using PAA binder demonstrated a large number of cracks over the whole electrode’s surface and severe pulverization of the SiMPs (Figure 15f, left). In addition to PAA, PEG-based binders can also be used for Si electrodes. The application of ureidopyrimidinone-functionalized polyethylene glycol (UPy-PEG-UPY) as a hydrogen-bonding self-healing binder led to excellent results (Figure 15g; Table 2, entry 8) [143]. The optimized composition of a UPy-PEG-Si-15 with 15 wt.% of Upy-PEG-UPY could heal spontaneously within 3 h (Figure 15h), while when using this binder, the initial Coulombic efficiency (ICE) was as high as 81% and a reversible capacity of 1454 mAh/g after 400 cycles corresponding to an average capacity decay of 0.04% per cycle was achieved. 

**Figure 16:**
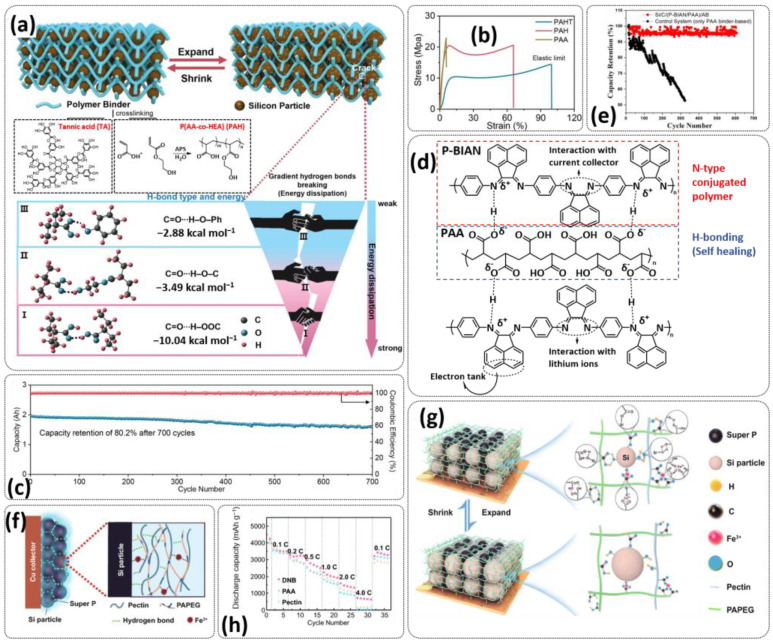
(**a**) Graphical representation depicting the expansion of the Si anode and the chemical structure of the gradient hydrogen-bonding binder (PAHT). Schematic illustration of the sequential energy-dissipation mechanism by this gradient H-bonding binder (PAHT) when the Si electrode expands. The hydrogen bond types and bond energies calculated by density functional theory simulations. The order of the gradient hydrogen bonds breaking is from weak to strong. (**b**) Stress–strain curves of the PAA, PAH and PAHT films. (**c**) Cycling performance of the NCM/Si-C full pouch cell at 1C between 2.75 and 4.2 V. Reproduced with permission from [149]. Copyright © 2021 Wiley-VCH GmbH. (**d**) Design strategy of the P-BIAN/PAA composite. (**e**) Comparison of the capacity retention of the Si/C/(P-BIAN/PAA)/AB anode with a control system with only PAA binder. Reproduced with permission from [146]. Copyright © 2022, American Chemical Society. (**f**) Design of the bioinspired double-network polymer binder DNB. (**g**) The supramolecular hybrid network in Si electrodes during cycling. (**h**) Rate capability of the Si (1.0 mg cm^−2^)/Li cells with different binders at different current rates. Reproduced from [147].

Inspired by the natural antifatigue titin protein, Hu et al. designed a gradient hydrogen-bonding polymer binder (PAHT) for Si-based anodes by incorporating tannic acid (TA) into poly(acrylic acid-*co*-2-hydroxyethyl acrylate) (PAH) copolymer (Figure 16a; Table 2, entry 14). For the preparation of PAHT binder, firstly, poly(acrylic acid-*co*-2-hydroxyethyl acrylate (PAH) with two types of polar groups (COOH and OH) was synthesized through a typical free radical polymerization reaction. The optimized composition containing 23.53 wt% PHEA displayed the lowest curvature and highest capacity retention. Subsequently, tannic acid (TA) with abundant polyphenol groups was introduced into the PAH to build a crosslinked network (PAHT) via multiple H-bonds between the polyphenol groups and COOH/OH of the PAH (mass ratio PAH:TA = 9:1). The well-defined gradient hydrogen bonds, with a successive bond energy of −2.88–−10.04 kcal mol^−1^, could effectively release the large stress of silicon via the sequential bonding cleavage (Figure 16a). This could avoid recurrently abrupt structure fracture of the traditional binder due to the lack of gradient energy dissipation. A stress–strain analysis revealed that PAH and PAHT films achieved over 66% and 103% strain before fracture, in contrast to PAA film, which displayed a brittle behavior with high a Young’s modulus (410 MPa) and low strain at break (6%) (Figure 16b). The PAHT binder endowed stable, high-areal capacity silicon-based electrodes > 4 mAh cm^−2^ and, more importantly, only a relatively low binder content of 4.2 wt% allowed for a very good cyclability of the battery. A gradient hydrogen-bonding binder was tested in a 2 Ah silicon-based pouch cell with an impressive capacity retention of 80.2% after 700 cycles (0.028% decay/cycle) (Figure 16c). Recently, Gupta et al. reported a highly robust n-type self-healing polymer composite poly(bisiminoacenaphthenequinone)/poly(acrylic acid) (P-BIAN/PAA) as a binder for Si anodes (Table 2, entry 11) [146]. The applied design concept is illustrated in Figure 16d. Combining P-BIAN as the conducting link with an ability to tailor a thin SEI with a protic polymer (PAA) to attain the self-healing property through hydrogen bond network formation led to an n-type self-healing binder which was evaluated to (i) provide mechanical robustness to the large volume expansion of Si particles, (ii) maintain electrical conductivity within the electrode laminate, and (iii) facilitate the formation of a thin SEI by restricting the extent of electrolyte decomposition on the surface of anode because of its low-lying lowest unoccupied molecular orbital (LUMO) that empowers its n-doping in the reducing environment. A conducted electrochemical evaluation confirmed the expectations. The Si/C/(P-BIAN/PAA)/AB anode showed an excellent cyclability up to 600 cycles, specific capacity of ∼2100 mAh g^−1^Si, ∼98.9% average Coulombic efficiency and 95% capacity retention after 600 cycles (Figure 16e). After the electrochemical evaluation of the Si/C/(P-BIAN/PAA)/AB anode, the postmortem analysis by XPS and SEM studies confirmed that the SEI formed in the case of the P-BIAN/PAA composite-based anode was thin, and the electrode morphology was nicely retained with good adherence to the current collector. Another example of the bioinspired Si anode binder is an endotenon sheath-inspired water-soluble double-network binder (DNB) (Table 2, entry 12) [147]. The concept of the design and self-healing principle of this binder is illustrated in Figure 16f,g). Mechanically robust DNB contains highly viscous pectin and the amphipathic copolymer PAPEG comprised of hydrophilic polyacrylic acid (PAA) and oleophilic polyethylene glycol diacrylate (PEGDA). In addition, ferric nitrate (Fe(NO_3_)_3_) was further introduced to construct coordinative bonds among the carboxylic acid units on the polymers for dissipating stress. The DNB could not only strongly glue Si particles by forming hydrogen bonding but could also form supramolecular interactions (e.g., hydrogen bonding, ion–dipole interactions and metal-coordination) between pectin and PAPEG to strengthen the adhesion structure. The formed supramolecular double network of DNB leads to stabilization of the electrode interface during volume shrinkage and expansion of the Si electrode upon cycling, thus minimizing the mechanical damage. Moreover, ferric nitrate can be reduced into Li_3_N during cycling, thus contributing to the formation of Li_3_N/LiF-rich SEI layer, which can suppress continuous electrolyte decomposition. DNB revealed superior electrochemical performance in Si/Li half cells and LiNi_0.8_Co_0.1_Mn_0.1_O_2_ (NCM811)/Si full cells. The capacity of Si/Li half cells with DNB binders could still remain at 1115 mAh g^−1^ after 300 cycles at a current density of 4.2 A g^−1^ (1 C), outperforming the traditional PAA and pectin binders (Figure 16h). Furthermore, NCM811 (11 mg cm^−2^)/Si full cells with DNB binder show good cycle performance, with a capacity retention of 86% at 0.1C after 50 cycles.

**Figure 17:**
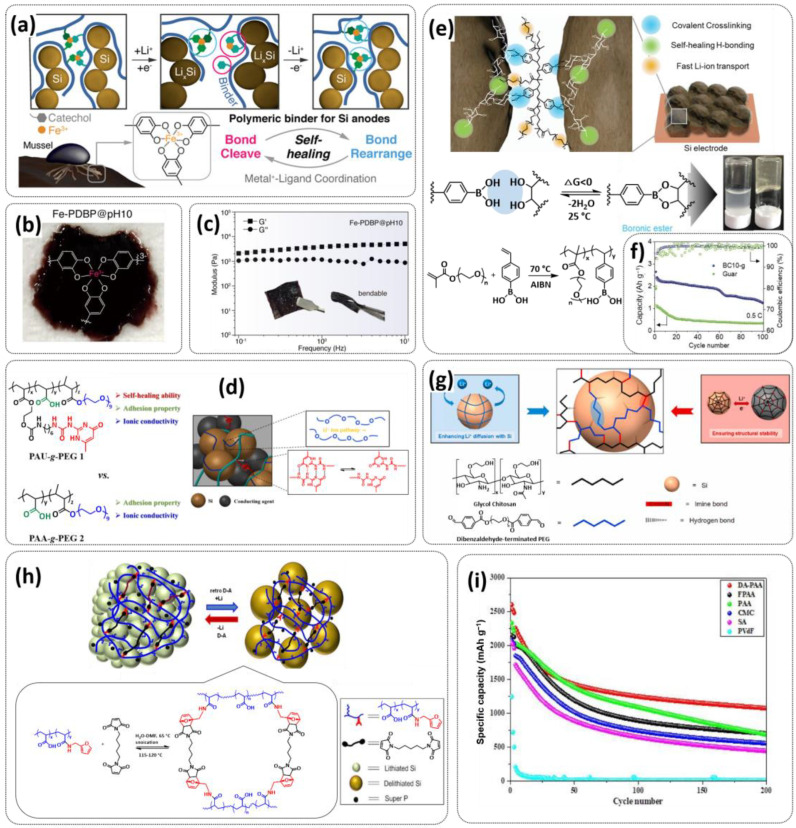
(**a**) Illustration of the self-healing mechanism of metallopolymer Fe–PDBP. (**b**) PDBP in the presence of FeCl_3_ at pH = 10. (**c**) Rheology of Fe–PDBP@pH10 in a frequency range from 10^−1^ to 10^1^ Hz when measured at a constant stress of 100 Pa (*G*′: elastic moduli; *G*′′: viscous moduli). Reproduced with permission from [145]. Copyright © 2019, American Chemical Society. (**d**) Schematic illustrations of the polymeric binder, PAU-*g*-PEG 1, with both reversible self-healing and Li^+^-conductive properties compared to PAA-g-PEG 2 with only Li^+^-conductive sites for silicon electrodes. Reproduced from [150]. (**e**) Synthesis of BC-g binders: Schematic of BC-g binder in the Si electrode, showing the formation of a covalent bond from BC, self-healing ability of abundant hydroxyl groups with PEO and boronic ester and fast Li-ion transport through PEO groups. The spontaneous crosslinking reaction of boronic acid and vicinal alcohol at room temperature along with pictures of guar (left) and BC-g binders (right). The synthesis process of BC. (**f**) Cycle retention of SiMP electrodes with guar and BC10-g binders at 0.5C for 100 cycles. Reproduced with permission from [144]. Copyright © 2019 WILEY-VCH Verlag GmbH & Co. KGaA, Weinheim. (**g**) Schematic illustration of the behavior of the xPEG-GCS binder in the silicon electrode. Reproduced with permission from [148]. Copyright © 2021 Elsevier B.V. All rights reserved. (**h**) Schematic representations of the self-healable polymeric binder based on reversible Diels–Alder chemistry. (**i**) The cycling performances of Si electrodes with various polymer binders, namely, DA-PAA, FPAA, PAA, CMC, SA, and PVdF, at a current density of 0.5C for 200 cycles. Reproduced with permission from [151]. Copyright © 2020 Elsevier Ltd. All rights reserved.

Jeong and Choi were inspired by mussels’ byssus to utilize a self-healing mechanism based on metal coordination to develop adaptive binders for Si electrodes (Figure 17a) [145]. They reported a copolymer binder with Fe^3+^–(tris)catechol coordination crosslinks, capable of forming a 3D supramolecular network by reversible metal–ligand coordination. To introduce the reversible Fe^3+^–(tris)catechol coordination bond in a network of flexible polymer chains, copolymers containing three different types of monomers were synthesized via radical polymerization (Table 2, entry 10). Dopamine methacrylamide (DMA) containing a catechol group was selected as the main monomer unit. In order to degrease the glass transition of rigid DMA polymer (T_g_ = 178.5 °C) and enable better wetting with conventional electrolytes, aliphatic butyl acrylate (BA) monomer (T_g_ = −50 °C) was introduced. As a third component, 1 mol % of polyethylene glycol diacrylate (PEGdA, *M*_n_ = 575) was added to serve as a permanent crosslinker and also to increase the electrolyte uptake. Once the copolymer was synthesized, FeCl_3_ was added to the PDBP solution in a molar ratio of catechol/Fe^3+^ = 3:1 to generate Fe^3+^–(tris)catechol coordination bonds as interchain crosslinks (Figure 17b). The rheological investigation revealed a gel-like behavior of the formed network (Figure 17c). The optimized Fe–PDBP@pH10 could heal after cutting within 24 h at room temperature. In cell tests, the Si electrode exhibited an 81.9% capacity retention after 350 cycles, which is far superior to the linear polymeric binders commonly used in LIBs. In order to introduce a strong hydrogen-bonding supramolecular network capable of compensating for the volume expansion of the Si anode while simultaneously enhancing the Li-ion conductivity, Kim and coworkers followed the approach presented in Figure 17d (Table 2, entry 15) [150]. A grafted copolymer poly(acrylic acid-*co*-UPy-acrylate)-*grafted*-PEG, PAU-*g*-PEG was prepared by grafting a ureido-pyrimidinone (UPy)-functionalized poly(acrylic acid) with poly(ethylene glycol)(PEG). While UPy-PAA polymer chains are responsible for the formation of the 3D supramolecular network over the quadrupole hydrogen bonding of UPy moieties and multiple carboxylic acid groups, the implemented PEG chains facilitate Li-ion transport, involving ion hopping over ether groups [26,165]. PAU-*g*-PEG showed a high adhesive force of 1.6 N, which is higher than that of PAA and of PAA-PEG without UPy groups, and this can be explained by the formation of a supramolecular network. The silicon electrode with the PAU-*g*-PEG copolymer as a binder retained a capacity of 1450 mAh g^−1^ at 350 cycles and a Coulombic efficiency of 99.4% even after 350 cycles, as well as a high capacity of 2500 mAh g^−1^ under a high current density of 3C.

Self-healing based on reversible, dynamic covalent bonds can also be an auspicious approach towards polymeric binders for Si anodes. Park and coworkers designed a room-temperature crosslinked natural polymer binder for Si anodes using an ionically conductive boronic crosslinker (BC) that spontaneously forms covalent bonds between vicinal alcohols of a commercial polysaccharide polymer (Guar gum) and boronic acid group conjugated to the stiff polystyrene backbone (Figure 17e; Table 2, entry 9) [144]. This enables the formation of a strong and dynamic covalent bonding supramolecular network without any external triggers. The other component in the crosslinker, polyethylene oxide, is responsible for the enhanced ionic conductivity of the polymer binder. Boronic crosslinked guar (BC-g) binder has a high mechanical strength with a network polymer structure, which is attributed to the well-balanced combination of secondary interactions and strong covalent bonding. The optimized binder, BC-10, contained polymer with a molecular weight of 10 kg mol^−1^ and 59% of the boronic acid. The BC10-g binder allowed for the fabrication of an extremely thick electrode with a high mass loading level of ≈2.1 mg cm^−2^ that corresponds to the areal capacity of ≈6 mA h cm^−2^, showing good cycling stability (Figure 17f). Another type of self-healing ion-conductive binder based on imine dynamic covalent bonds was presented by Nam et al. (Table 2, entry 13) [148]. The self-healing conductive binder xPEG-GCS was prepared by mixing of glycol chitosan (GCS) polymer matrix with dialdehyde-terminated polyethylene glycol as a ion conducting macro-crosslinker (Figure 17g). The formation of strong and reversible imine double bonds in the crosslinked polymer provides the self-healing ability, while the PEG crosslinker improves the ion conductivity. Si electrodes using the developed polymer network results in an initial Coulombic efficiency of 82.2%, a discharge capacity of 2141 mAh g^−1^ after 150 cycles and a reversible capacity of 2700 mAh g^−1^ at a current density of 3C. Although the last two examples can actually be classified as hydrogels, they are mentioned here in order to highlight the possibility of the implementation of reversible imine and borate bonds for the preparation of self-healing binders for Si anodes.

Diels–Alder (D−A) chemistry was implemented in PAA-based self-healing binders by Rejeev et al. (Table 2, entry 16) [151]. In this study, 1,6-bismaleimide (BMI) was added as a crosslinker to furfurylamine-functionalized poly(acrylic acid) (FPAA) to form a dynamic 3D crosslinked polymer network based on reversible thermal Diels–Alder (D–A) click chemistry (Figure 17h). The formation of the D−A 3D network occurs at 65 °C, whereas at 120 °C the gel becomes liquid, indicating the reversible cleavage of D-A bonds. The Si electrode with the D-A-adduct binder (DA-PAA) had excellent adhesive strength compared to conventional binders, such as poylacrylic acid (PAA), carboxymethyl cellulose (CMC), sodium alginate (SA) and poly(vinylidene fluoride) (PVdF). The Si electrode with the DA-PAA polymer (Si@DA-PAA), had an initial discharge capacity of 2607 mAh g^−1^ and the highest specific capacity (1076 mAh g^−1^) among all of the electrodes tested of after charging and discharging over the course of 200 cycles at a current density of 0.5C (Figure 17i). 

### 4.2. Self-Healing Binders for Li-Metal Anodes

**Figure 18:**
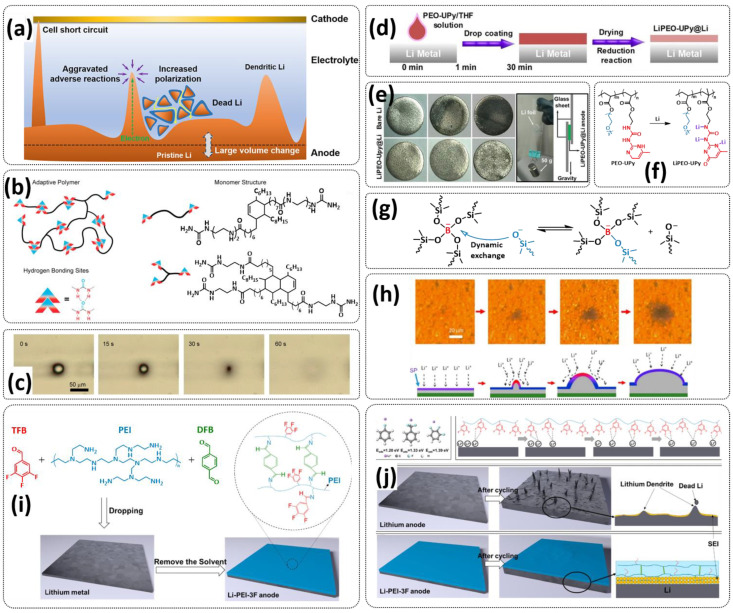
(**a**) Scheme of dilemma for Li-metal anode in rechargeable batteries. Reproduced with permission from [166]. Copyright © 2017, American Chemical Society. (**b**) Chemical structure of the SHP. The black lines are the fatty acid backbone, and the red–blue boxes are the urea hydrogen-bonding sites. The right-hand side shows the molecular structure of the diacid and triacid backbones. (**c**) Optical microscope images showing the flowability of the polymer coating via the healing process of an artificially created pinhole. Reproduced with permission from [152]. Copyright © 2016, American Chemical Society. (**d**) Schematic diagram of PEO–UPy coating on Li-metal surface. (**e**) Photographs of bare Li and LiPEO–UPy@Li anodes exposed to ambient air for different durations (**left**) and adhesion test of PEO–UPy@Li anode against 50 g mass (**right**). (**f**) Chemical structures of the PEO-UPy and LiPEO-UPy polymers. Reproduced with permission from [155]. Copyright © 2020 Wiley-VCH Verlag GmbH & Co. KGaA, Weinheim. (**g**) Schematic diagrams showing the design of the SP-modified Li anode. (**h**) Series of optical microscope images depicting the Li-metal deposition process on SP-coated Cu. Scale bar: 20 μm. (**above**) Schematic of Li deposited on SP-coated Cu. The red color in the SP coating represents the local dynamic stiffening. (**bottom**) Reproduced with permission from [130]. Copyright © 2017, American Chemical Society. (**i**) Schematic diagrams of the preparation of the PEI-3F interlayer on a Li-metal anode. (**j**) Theoretical calculations on the adsorption binding energies between lithium ions and the F-containing groups; Schematic diagram of the influence of the molecular motion of the grafted F groups on the distribution of Li^+^ at the electrolyte/electrode interface. Li-deposition process on the bare Li (**up**) and Li-PEI-3F anodes (**bottom**). Reproduced with permission from [156]. Copyright © 2021, American Chemical Society.

Since Li-metal possesses a high theoretical capacity (3860 mAh g^−1^ or 2061 mAh cm^−3^) and the lowest electrochemical potential (−3.04 V vs. the standard hydrogen electrode), Li-metal batteries are regarded as the most promising alternative for high-energy density advanced battery systems. The primary technical problems impeding the practical application of Li-metal anodes include high reactivity, ample dendrite formation, huge volume fluctuations, poor cycling performance and safety issues [134,166]. In general, during charging/discharging Li deposits as dendrites (Figure 18a), which can at the distinct point damage the separator membrane between electrodes, causing an internal short circuit of the cell, leading to the thermal runaway and explosion hazards. Thus, the suppression of Li-dendrite formation is one of the major issues. The solid electrolyte interface (SEI) layer is a crucial factors in preventing side reactions between electrolyte and lithium metal, thus suppressing Li-dendrites. However, repetitive volume changes and deleterious reactions between electrodes and electrolytes during cycling cause generate an unstable and thick SEI, while consuming more Li-ion and electrolytes for continuous “re-formation” of SEI. This leads to a decrease in the Coulombic efficiency (CE) and a continuous increase in the Li-metal anode overpotential, resulting in a reduction of the battery’s capacity and lifetime. Thus, the formation of a stable SEI layer on the Li-metal anode that is able to compensate for the large volume changes during cycling is the biggest challenge. There have been various approaches to compensate for these drawbacks [34,35,166]; we focus here on the application of self-healing polymers as protective binders. 

Bao and coworkers used their previously developed SHB for a Si anode [24], as well as for an adaptive binder on a lithium metal anode (Figure 18b; Table 2, entry 17) [152]. The mechanical properties of the binder depend strongly on the content of the hydrogen bonding urea groups, while the electrochemical properties seem to be insensitive towards variations in the composition. The optimized SHP with a 16:80 ratio (Table 2, entry 17) exhibited efficient self-healing (Figure 18c). At a high current density of 5 mA/cm^2^, a flat and dense lithium metal layer was obtained, and stable cycling Coulombic efficiency of ∼97% was maintained for more than 180 cycles at a current density of 1 mA/cm^2^. Wang et al. presented an adhesive and self-healable supramolecular copolymer, comprising of pendant poly(ethylene oxide) segments and ureido-pyrimidinone (UPy) hydrogen-bonding moieties as a protection layer of a Li anode (Figure 18d; Table 2, entry 21) [155]. Due to the formation of the supramolecular network via the quadrupole hydrogen bonding of UPy groups, a robust artificial protective layer was formed (Figure 18e). The protective layer adhered firmly on the Li surface to achieve long-term Li plating/stripping cycling at high current densities and high areal capacity. The protection performance of in situ formed LiPEO–UPy SEI layer was significantly enhanced owing to the strong binding and improved stability arising from a spontaneous reaction between UPy groups and Li-metal (Figure 18f), which was confirmed by X-ray photoelectron spectroscopy (XPS) depth profiling analysis and nuclear magnetic resonance (^1^H NMR) spectroscopy. An ultrathin (approximately 70 nm) LiPEO–UPy layer can contribute to stable and dendrite free cycling at a high areal capacity of 10 mAh cm^−2^ at 5 mA cm^−2^ for 1000 h. 

In addition to hydrogen-bonding-based supramolecular networks, self-healing polymers based on dynamic covalent bonds have also been extensively studied for application as binders on Li-metal anodes. An adaptive dynamically crosslinked polymer “solid-liquid” interfacial protective layer for Li-metal anodes was designed by Cui and coworkers (Table 2, entry 18) [130]. Thereby polydimethylsiloxane (PDMS) was dynamically crosslinked by transient boron-mediated crosslinks (Figure 18g), resulting in a shear-thickening material SP. This “solid-liquid” property originate from the constant “breaking-reconnecting” behavior of the dynamic borate crosslinks, allowing the material to increase its stiffness with increasing stress (e.g., shear rate and stretching rate). Thus, the properties of flowability and stiffness of the SP can reversibly switch depending on the rate of lithium growth. Fluidlike SP can homogenously spread on the Li-metal anode. When a local inhomogeneity in Li deposition rate occurs on the electrode, the SP will respond and stiffen to constrain the localized increase in the Li growth. However, once the aberrant growth rate is suppressed by the SP, the coating recovers its fluidlike property and conformally adapts its shape according to the structure of the lithium metal electrode (Figure 18h). A full cell with SP-coated Li-metal anode combined with LiFePO_4_ (LFP) cathode maintained a high average Coulombic efficiency of 99.5% and a stable capacity of 142.1 mAh/g for over 50 cycles. Cui et al. fabricated a self-healing interlayer composed of a trifluorophenyl-modified poly(ethylene imine) network crosslinked by dynamic imine bonding (PEI-3F) (Figure 18i; Table 2, entry 22) [156]. The trifluorophenyl moieties of the interlayer can coordinate with Li^+^, which enables the interlayer to adjust the distribution of Li^+^ at the electrode/electrolyte interface, while the imine bonding endows the interlayer with self-healing capability. The formed SEI layer can be in situ-embedded on the PEI interlayer during the initial charging and discharging processes and autonomously recover itself via the self-healing of the PEI-3F interlayer after being pierced by the lithium dendrites (Figure 18j).

**Figure 19:**
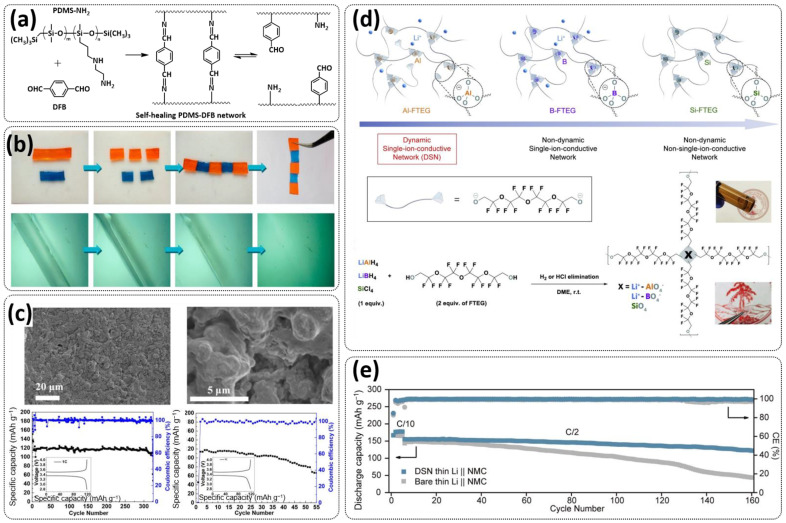
(**a**) Synthesis of a self-healing PDMS-DFB network. (**b**) Optical and optical microscopic images of the self-healing of the PDMS-DFB elastomer under ambient conditions (25 °C). (**c**) (**above**) SEM images of the Li deposits on Cu-PDMS electrodes after 50 cycles of deposition/stripping with 1 mAh cm^−2^ at 0.5 mA cm^−2^. (**below**) Cycling performance of Li/LiFePO_4_ full cells comprising the PDMS-DFB-coated Li-metal anode (**left**) and bare Li-metal anode (**right**). The anode was predeposited on 5 mAh cm^−2^ Li-metal and tested at 0.2C for the first cycle and 1.0C for the later cycles. The insets are the charge–discharge profiles of the corresponding cells. Reproduced with permission from [153]. Copyright © 2018, American Chemical Society. (**d**) Material design and chemical structures of the DSN and derivatives. (**e**) Long-term cycling performance of the DSN thin Li||NMC532 (cyan) and bare thin Li||NMC532 (gray) full batteries. Reproduced with permission from [154]. Copyright © 2019 Published by Elsevier Inc.

Cui et al. reported the stabilization of the SEi layer by a self-healable polydimethylsiloxane (PDMS) network crosslinked via dynamic covalent imine bonding [153]. A self-healing flexible PDMS-DFB elastomer was synthesized through a Schiff base reaction by simply mixing 1,4-diformylbenzene (DFB) and xiameter OFX-8040A fluid (PDMS-NH_2_) (Figure 19a; Table 2, entry 19). The obtained elastomer exhibited self-healing properties through the transamination between −C═N– and −NH_2_ in the dynamic imine covalent network, and could fully heal in 3 min under ambient conditions (Figure 19b). Additionally, the PDMS-DFB elastomer was also self-healable in an organic electrolyte such as 1.0 mol L^−1^ LiTFSI/DOL-DME. The application of the strategy was demonstrated by a Li/LiFePO_4_ full battery that exhibited a capacity retention up to 99% and a Coulombic efficiency > 99.5% after 300 cycles (Figure 19c). Bao and coworkers presented a dynamic single-ion-conductive network (DSN) as a multifunctional artificial SEI [154]. An artificial SEI was designed in order to integrate all requirements in a single matrix, enabling an ideal interface by regulating critical features, such as fast ion transport, conformal protection, and parasitic reaction mitigation. The DSN (Figure 19d; Table 2, entry 20) incorporates the tetrahedral Al(OR)_4_^−^ (R = soft fluorinated linker) centers as both dynamic bonding motifs and counter anions, endowing it with flowability and Li^+^ single-ion conductivity. Simultaneously, the nonreactive fluorinated linkers (1H,1H,11H,11H-perfluoro-3,6,9-trioxaundecane-1,11-diol, FTEG) provide chain mobility and electrolyte-blocking capability. The Li^+^ counter ions are introduced as the mobile ions in the network. The reversibility and applicability of the self-healing materials with Al-O dynamic bonds was studied for the first time. The DSN shows an ion conductivity of 3.5 ± 2.3 × 10^−5^ S cm^−1^ without addition of salt or electrolyte, which is essentially high for interfacial ion conduction. The DSN coating greatly decreases impedance while increasing the rate capability of Li||NMC full cells, obtaining a discharge capacity of 132.9 mAh g^−1^ compared to 88.2 mAh g^−1^ for the uncoated anodes at a rate of 1C. Given a higher average CE (>99.6%), the DSN Li||NMC full cell also showed a much improved capacity retention of ∼85% after 160 cycles at a charge–discharge rate of C/2 compared to that of the bare Li cell (<30% retention) (Figure 19e). 

## 5. Conclusions

We have discussed the different approaches to designing self-healing polymers suitable for implementation in lithium batteries either as electrolytes or as adaptive binders for electrodes. Table 1 and Table 2 provide a comprehensive summary of the different concepts for the material design, self-healing mechanism and conditions and electrochemical performance of polymer electrolytes and binders. In conclusion, self-healing polymers implemented in electrolytes or electrodes may be able to optimize the cycle stability and prolong the lifetime of the batteries, while simultaneously improving the safety. However, research in this field is still in its initial stage and far from actual commercialization. The requirements of the materials are enormous, and further research needs to be performed to develop materials with desired properties, such as a high mechanical and chemical stability, improved ionic conductivity, sufficient dialectic constant and enhanced elasticity. As electrolytes, self-healing polymers should exhibit enhanced Li-solvation properties (dialectic constant), preventing Li-clustering and, thus, increasing the ion conductivity. Hereby, the crystallinity, T_g_ and Li^+^-transport properties of the polymer matrix, as well as the influence of the self-healing mechanism on Li-diffusion, play a crucial role. Furthermore, a good compatibility of the electrode materials should be provided to ensure the formation of a stable SEI layer, thus prolonging the cycling stability of the battery. For the binders on Si anodes, self-healing polymer networks are needed that are strong and flexible enough to compensate for the enormous volume changes (>300%) during the charging–discharging of the battery. Additionally, the ion conductivity of the binder should be ensured in order to enable effective Li-diffusion to the electrode. Regarding Li-metal anodes, a crucial issue is connected to the suppression of Li-dendrite growth on the anode. Ideally, the self-healing polymer should possess sufficient mechanical strength and flexibility to suppress Li-dendrite growth while being able to chemically stabilize the SEI layer. Overall, further understanding and development of self-healable materials are necessary both in the field of fundamental research and industrial application to ensure the performance improvements of novel materials and simultaneously their compatibility with each other. 

## Figures and Tables

**Table 1:** Various self-healing polymers evaluated as electrolytes in lithium batteries.

Entry	Self-Healing Component/Electrolyte	Type	Healing Mechanism	Battery/Additives	Healing Conditions	Performance/Ionic Conductivity	Reference
1	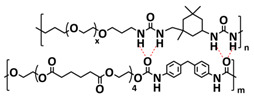	SPE	H-bonds	LMB, Li/SHSPE/NCM, 2 M LiClO_4_ (or LiTFSI or LiBF_4_) in DEC/DMC/EC	RT, 60 s	170.6 mA h g^−1^ at 0.1C, σ (RT) = 1.72 × 10^−5^ S cm^−1^	[70]
2	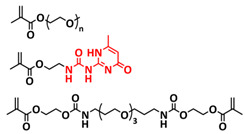	SPE	H-bonds	LMB, LiFePO_4_/DN-SHPE/Li, LITFSI (EO/Li^+^ molar ratio 16:1)	60 °C, 2 h	137.9 mA h g^−1^ at 0.1C, σ (30 °C) = 1.72 × 10^−5^ S cm^−1^	[71]
3	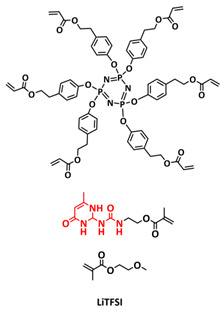	SPE	H-bonds	LMB, Li/CPSHPE1/LFP, LiTFSI (EO/Li^+^ molar ratio 16:1)	60 °C, 3 h	130 mA h g^−1^ at 0.1C, σ (30 °C) = 8.9 × 10^−5^ S cm^−1^	[72]
4	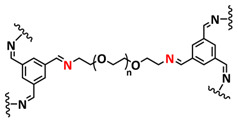	SPE	Imine dynamic covalent bonds	LMB, Li|SHSPE| LiFePO_4_, LiPF_6_ (EO/Li^+^ molar ratio 10:1)	RT, 24 h	156 mA h g^−1^ at 0.1C, σ (RT) = 7.48 × 10^−4^ S cm^−1^	[73]
5	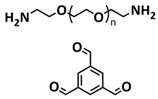	SPE	Imine dynamic covalent bonds	LMB, LFP/PBPE/Li, 0.8 M LiTFSI + 0.3 M LiDFOB + 0.0.2 M LiPF_6_ in EC/FEC/EPC (75:25:5 vol)	60 °C, 0.5 h	158 mA h g^−1^ at 0.1C, σ (RT) = 4.79 × 10^−3^ S cm^−1^	[74]
6	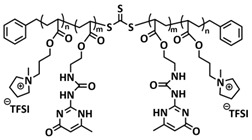	SPE	H-bonds, ionic interactions	NA	40 °C, 1 h	NA, σ (30 °C) = 4.4 × 10^−5^ S cm^−1^	[49]
7	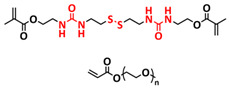	SPE	H-bonds, disulfide dynamic covalent bonds	LIB and LMB, Li|3PEG-SSH| LFP, LiTFSI (EO/Li^+^ molar ratio 16:1)	RT, 30 min	140 mA h g^−1^ at 0.1C, σ (RT) = 7.28 × 10^−6^ S cm^−1^	[75]
8	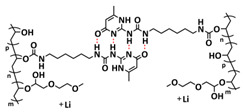	SPE	H-bonds	LMB, Li/PVA-UPy-PEG750/LFP, LiClO_4_ (EO/Li^+^ molar ratio 11:1)	60 °C, 1 h	145 mA h g^−1^ at 0.1C, σ (60 °C) = 1.5 × 10^−4^ S cm^−1^	[76]
9	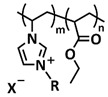	SPE	Ionic interactions	NA	55 °C, 7.5 h	NA, σ (RT) = 1.6 × 10^−7^ S cm^−1^	[52]
10	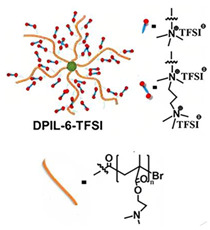	SPE	Ionic interactions	LIB, LFP/SPE/Li, EMIMTFSI, LiTFSI	RT, <2 h	153 mA h g^−1^ at 0.1C, σ (RT) = 5.5 × 10^−5^ S cm^−1^	[77]
11	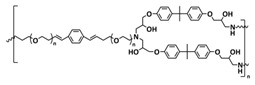	SPE	Imine dynamic covalent bonds	LMB, Li|ShSPE-3|LiFePO_4_, LiTFSI (EO/Li^+^ molar ratio 8/16/24)	RT, 30 min	141 mA h g^−1^ at 0.1C, σ (60 °C) = 1.7 × 10^−4^ S cm^−1^	[78]
12	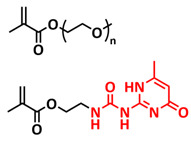	SPE	H-bonds	LIB, LFP/shPE/Li, LiTFSI (EO/Li^+^ molar ratio 20:1)	RT, 2 h	155 mA h g^−1^ at 0.1C, σ (60 °C) = 1.1 × 10^−4^ S cm^−1^	[79]
13	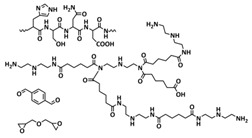	SPE	Imine dynamic covalent bonds, H-bonds	LIB and LMB, Li|SPI-3Li|LiFePO_4_, LiTFSI	100 °C or hot water	32.6 mA h g^−1^ at 0.2C, σ (30 °C) = 3.3 × 10^−4^ S cm^−1^	[80]
14	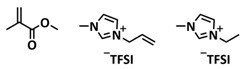	SPE	Ionic interactions	LMB, Li/EMIMTFSI/P (MMA-co-AMIMTFSI)-40/LFP, LiTFSI	RT, 5 min	135 mA h g^−1^ at 0.1C, σ (RT) = 1.9 × 10^−4^ S cm^−1^	[81]
15	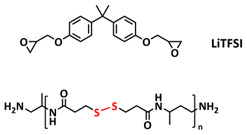	SPE	Disulfide dynamic covalent bonds, ionic interactions	NA, LiTFSI 10–20 wt%.	100 °C, 1 h	NA, σ (80 °C) = 3.3 × 10^−6^ S cm^−1^	[82]
16	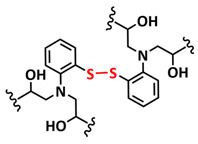	SPE	Disulfide dynamic covalent bonds, H-bonds	LMB, LiFePO_4_/RFSPE-3/Li	RT, 1 h	96 mA h g^−1^ at 0.2C, σ (RT) = 2.1 × 10^−3^ S cm^−1^	[83]
17	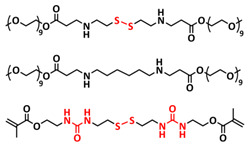	SPE	Disulfide dynamic covalent bonds, H-bonds	LMB, Li/PESS20/LiFePO_4_, LiClO_4_ (EO/Li^+^ molar ratio 16:1)	80 °C, 2 h	138.2 mA h g^−1^ at 0.1C, σ (30 °C) = 1.2 × 10^−4^ S cm^−1^	[84]
18	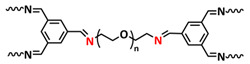	SPE	Imine dynamic covalent bonds	LMB, Li|shCLSPE-3400|LiFePO_4_, LiTFSI (EO/Li^+^ molar ratio 20:1)	RT, 10 min	141.6 mA h g^−1^ at 0.1C, σ (RT) = 4.8 × 10^−4^ S cm^−1^	[85]
19	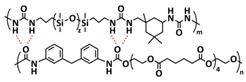	SPE	H-bonds	LMB, 2 M LiClO_4_ (or LiTFSI or LiBF_4_)	RT, 3 min	NA, σ (RT) = 2.5 × 10^−4^ S cm^−1^	[86]
20	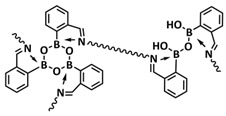	SPE	Borate and imine dynamic covalent bonds	LMB, LFP/IBshPE/Li, 0.02 M LiPF_6_ + 0.3 M LiDFOB + 0.8 M LiFSI in FEC/EC (30:70 vol)	RT, 4 h	130.5 mA h g^−1^ at 2C, σ (30 °C) = 5 × 10^−3^ S cm^−1^	[87]
21	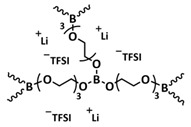	SPE	Borate dynamic covalent bonds	NA, LITFSI	60 °C, 34 h	NA, σ (90 °C) = 3.5 × 10^−4^ S cm^−1^	[88]
22	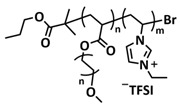 + PEO + LITFSI	SPE	Ionic interactions	LIB and LMB, Li/PEO@ BPIL-3-SPE/LiFePO_4_, LiTFSI 10%wt.	60 °C, <30 min	163 mA h g^−1^ at 0.2C, σ (30 °C) = 1 × 10^−5^ S cm^−1^	[89]
23	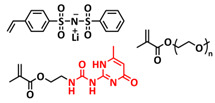	SIPE	H-bonds, ionic interactions	LMB, Li/SIGPE-5 membrane/LFP, PC	60 °C, 12 h	129 mA h g^−1^ at 0.1C, σ (60 °C) = 1.4 × 10^−5^ S cm^−1^	[90]
24	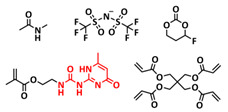	DSP	H-bonds	LMB, Li/DSP/LiMn_2_O_4_, LiTFSI, FEC, NMA	RT, 1 h	117 mA h g^−1^ at 0.1C, σ (RT) = 1.79 × 10^−3^ S cm^−1^	[91]
25	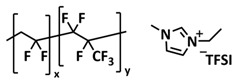	GPE	Ion–dipole interaction	LMB, LiFePO_4_/LiTFSI-IL-P (VDF-HFP)/Li, 1 M LiTFSI in DME/DOL	RT, 12 h	145 mA h g^−1^ at 0.2C, σ (RT) = 8.8 × 10^−4^ S cm^−1^	[92]
26	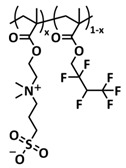	GPE	Ion–dipole interaction	LIB, Li/SHSPE3/LiFePO_4_, EMIMTFSI 25 wt.%, LiTFSI	60 °C, 10 min	145 mA h g^−1^ at 0.2C, σ (60 °C) = 5 × 10^−4^ S cm^−1^	[93]
27	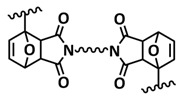	GPE	Diels–Adler reaction, ion–dipole interaction	LIB, EMIMTFSI	100 °C, 20 min	NA, σ (RT) = 3.3 × 10^−3^ S cm^−1^	[94]
28	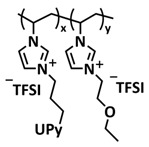	GPE	H-bonds, ionic interactions	LIB, Li|ionogel|LiFePO_4_, DE-IM/TFSI, LiTFSI	55 °C, 1 h	147.5 mA h g^−1^ at 0.2C, σ (RT) = 1.5 × 10^−3^ S cm^−1^	[95]
29	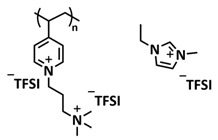	GPE	Ionic interactions	LMB, Li/PVT-45%EMIMTFSI/LiFePO_4_, LiTFSI	RT, <1 h	145 mA h g^−1^ at 0.1C, σ (RT) = 1.3 × 10^−4^ S cm^−1^	[96]
30	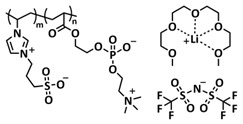	GPE	Ionic interactions	LIB and LMB, graphite|f-ZI solvate ionogel|NCM	55 °C, 1 h	140 mA h g^−1^ at 0.1C, σ (RT) = 6 × 10^−4^ S cm^−1^	[97]
31	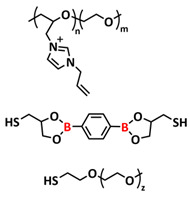	GPE	Borate dynamic covalent bonds, ionic interactions	NA	120 °C, 2 h	NA, σ (30 °C) = 1.6 × 10^−5^ S cm^−1^	[98]
32	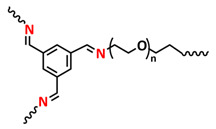	GPE	Imine dynamic covalent bonds	LMB, LiFePO_4_/IGPE-50/Li, 0.1 M LiTFSI in BMImTFSI	RT, 10 s	155 mA h g^−1^ at 0.1C, σ (5 °C) = 3.1 × 10^−4^ S cm^−1^	[99]
33	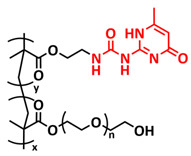	GPE	H-bonds	LIB, Li/GPE-20/LFP, EMIMTFSI	RT, 15 h	161.5 mA h g^−1^ at 0.05C, σ (RT) = 7.5 × 10^−4^ S cm^−1^	[100]
34	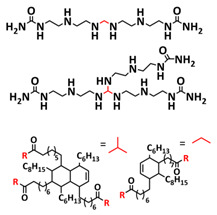	CPE	H-bonds	LIB, Li/CPE/Li_4_Ti_5_O_12_, 1 M LiPF_6_ in EC/DEC	RT, 1 h	157 mA h g^−1^ at 0.2 C; NA	[101]
35	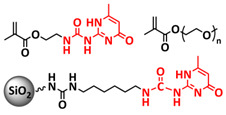 + LiTFSI	CPE	H-bonds	LIB, Li|SHCPE-10|LFP, LiTFSI (EO/Li^+^ molar ratio 16:1)	RT, 1 h	145 mA h g^−1^ at 0.2C, σ (30 °C) = 8 × 10^−4^ S cm^−1^	[102]
36	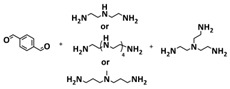 + Li_2_S-P_2_S_5_	CPM	Imine dynamic covalent bonds	LIB, FeS_2_/CPM/Li	NA	450 mA h g^−1^ at 0.2C, σ (30 °C) = 1 × 10^−4^ S cm^−1^	[103,104]
37	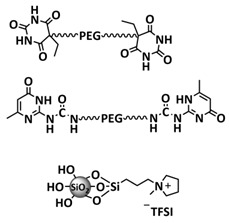	CPE	H-bonds	LIB, LITFSI (EO/Li^+^ molar ratio 5:1)	NA	NA, σ (80 °C) = 1 × 10^−3^ S cm^−1^	[105]
38	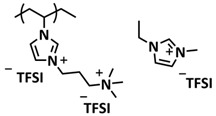 + BN nanosheets + LiTFSI	CPE	Ionic interactions	LMB, LFP/PolyIL-5/Li, LiTFSI	RT, 1 h	152 mA h g^−1^ at 0.1C, σ (RT) = 1.6 × 10^−4^ S cm^−1^	[106]

RT: room temperature; SPE: solid polymer electrolyte; LMB: lithium metal battery; NCM: LiNi_0.6_Co_0.2_Mn_0.2_O_2_; σ: ion conductivity; LiClO_4_: lithium perchlorate; LiTFSI: lithium bis(trifluoromethanesulfonyl)imide; LiBF_4_: lithium tetrafluoroborate; EC: ethylene carbonate; DEC: diethyl carbonate; DMC: dimethyl carbonate; EO: ethylene oxide; LiPF_6_: lithium hexafluoro phosphate; LFP: LiFePO_4_; LiDFOB: lithium difluoro(oxalato)borate; FEC: fluoroethylene carbonate; EPC: ethoxy (pentafluoro) cyclotriphosphazene; NA: not available; LIB: lithium-ion battery; DPIL-6-TFSI: six-armed dicationic ionic liquid with TFSI anion; EMIMTFSI: 1-ethyl-3-methylimidazolium bis(trifluoromethanesulfonyl)imide; TFSI: bis(trifluoromethanesulfonyl)imide anion; LiFSI: lithium bis(fluorosulfonyl)imide; PEO: poly(ethylene glycol)/poly(ethylene oxide); SIPE: single-ion conducting polymer electrolyte; PC: propylene carbonate; DSP: deep eutectic solvent (DES)-based self-healing polymer; NMA: N-metyhlacetamide; GPE: gel polymer electrolyte; DME: 1,2-dimethoxyethane; DOL: 1,3-dioxolane; DE-IM/TFSI: 1,2-dimethyl-3-etoxyethyl imidazolium bis(trifluoromethanesulfonyl)imide; BMImTFSI: 1-butyl-3-methyl imidazolium bis(trifluoromethanesulfonyl)imide; CPE: composite polymer electrolyte; CPM: composite polymer membrane; BN: boron nitride.

**Table 2:** Various self-healing polymers evaluated as binders for Si- and Li-metal anodes.

Entry	Self-Healing Binder	Healing Mechanism	Battery	Healing Conditions	Electrode/Electrolyte	Performance	Reference
1	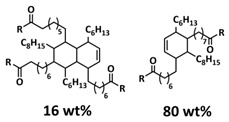	H-bonds	LIB	RT	Si ^a^/1 M LiPF_6_ in EC/DEC/FEC	Particle size 3–8 µm, 2617 mA h g^−1^ at 0.4 C; 80% after 90 cycles	[24]
2	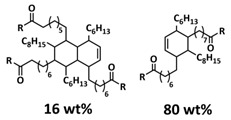	H-bonds	LIB	RT	Si ^a^/1 M LiPF_6_ in EC/DEC/VC/FEC	Particle size 500 nm-1.5 µm, 2620 mA h g^−1^ at 0.1 C; 80% after 500 cycles	[137]
3	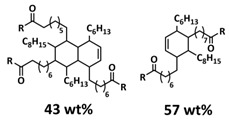	H-bonds	LIB	-	Si ^a^/1 M LiPF_6_ in EC/DEC/VC/FEC	Particle size 800 nm, 1700 mA h g^−1^ at 0.1 C; 80% after 178 cycles	[138]
4	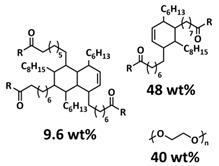	H-bonds	LIB	RT, 3 h	Si ^a^/1 M LiPF_6_ in EC/DEC/FEC	Particle size 800 nm, 1300 mA h g^−1^ at 0.5 C; 80% after 150 cycles	[139]
5	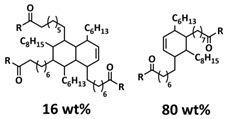	H-bonds	LIB	RT	Si ^b^/1 M LiPF_6_ in EC/FEC	Particle size 1–5 µm, 2212 mA h g^−1^ at 0.1 C; 92% after 100 cycles	[140]
6	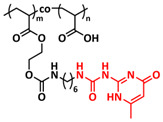 PAA -UPY	H-bonds	LIB	RT	Si ^c^	Particle size 70 nm, 4194 mA h g^−1^ at 0.5 C; 63% after 110 cycles	[141]
7	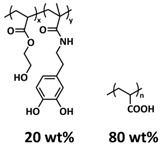 PAA-P(HEA-co-DMA)	H-bonds	LIB	RT	Si ^c^/1 M LiPF_6_ in DMC/EC/FEC	Particle size 2–6 µm, 2850 mA h g^−1^ at 0.4 C; 99.7% after 110 cycles	[142]
8	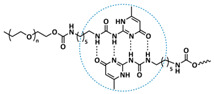 UPy-PEG-UPy	H-Bonds	LIB	RT	Si ^c^/1 M LiPF_6_ in DMC/EC	Particle size 50–70 nm, 1847 mA h g^−1^ at 0.4 C; 79% after 400 cycles	[143]
9	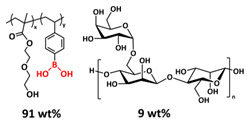	H-bonds, borate ester bonds	LIB	RT, 3 h	Si ^c^/1.3 M LiPF_6_ in DEC/EC	Particle size 50 nm, 2750 mA h g^−1^ at 0.2 C; 87% after 100 cycles	[144]
10	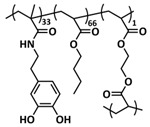 Poly(DMA-BA-PEGda) = PDBP	Metal coordination	LIB	RT, 24 h	Si ^c^/1 M LiPF_6_ in DMC/EC	Particle size 50 nm; 82% after 350 cycles at 1 C	[145]
11	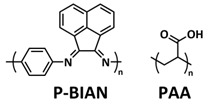	H-bonds	LIB	RT	Si ^c^/1 M LiPF_6_ in EC/DEC	2100 mA h g^−1^ at 500 mA g^−1^; 95% after 600 cycles	[146]
12	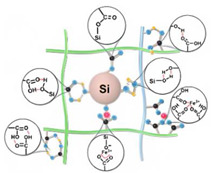	H-bonds, metal coordination	LIB	RT	Si ^c^/1 M LiPF_6_ in EC/DEC	1115 mA h g^−1^ at 1 C; 86% after 50 cycles	[147]
13	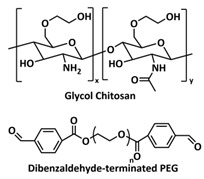	Amine dynamic covalent bonds	LIB	RT	Si ^c^/1 M LiPF_6_ in EC/DMC/FEC	2141 mA h g^−1^ at 0.5 C; 65% after 150 cycles	[148]
14	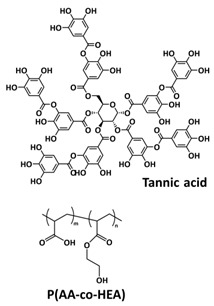	H-bonds	LIB	RT	Si ^c^/1 M LiPF_6_ in EC/DEC	1976 mA h at 1 C; 80% after 700 cycles	[149]
15	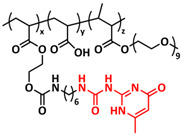 PAA-g-PEG	H-bonds	LIB	RT	Si ^c^/1 M LiPF_6_ in EC/EMC/FEC	1450 mA h g^−1^ at 0.5 C; 99% after 350 cycles	[150]
16	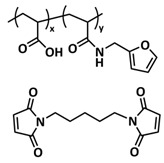	Diels–Adler reaction	LIB	65 °C, 1 h	Si ^c^/1 M LiPF_6_ in EC/EMC/FEC	1076 mA h g^−1^ at 0.5 C; 99.7% after 200 cycles	[151]
17	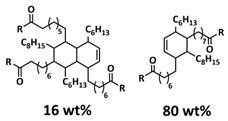	H-bonds	LMB	RT, 60 s	Li ^d^/1 M LiTFSI in DOL/DME/LiNO_3_	2617 mA h g^−1^ at 0.4 A g^−1^; 80% after 90 cycles	[152]
18	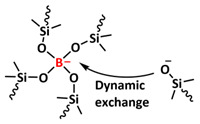	Borate dynamic covalent bonds	LMB	RT, 1 h	Li ^e^/1 M LiTFSI in DOL/DME	1 mA h cm^−2^ at 0.5 mA cm^−2^ for 120 cycles	[130]
19	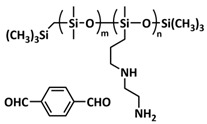 PDMS-DFB	Imine dynamic covalent bonds	LMB	RT, 12 h	Li ^f^/1 M LiTFSI in DOL/DME/LiNO_3_	1 mA h cm^−2^ at 0.5 mA cm^−2^ for 120 cycles	[153]
20	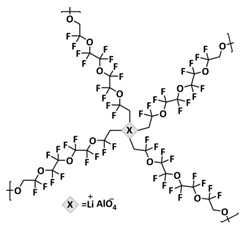	Al-O dynamic covalent bonds	LMB	RT, 12 h	Li ^g^/1 M LiPF_6_ in EC/DEC/FEC	1 mA h cm^−2^ at 1 mA cm^−2^ for 250 cycles	[154]
21	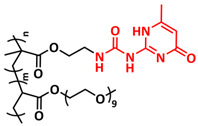 PEO-UPy	H-bonds	LMB	RT	Li ^h^/1 M LiPF_6_ in EC/FEC	10 mA h cm^−2^ at 5 mA cm^−2^ for 1000 cycles	[155]
22	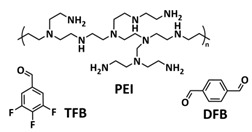	Amine dynamic covalent bonds	LMB	RT	Li ^h^/1 M LiTFSI in DOL/DME	1 mA h cm^−2^ at 1 mA cm^−2^ for 250 cycles	[156]

RT: room temperature; Si: silicon; LIB: lithium-ion battery; LMB: lithium metal battery; LiPF_6_: lithium hexafluorophosphate; LITFSI: lithium bis(trifluoromethanesulfonyl)imide; TFSI: bis(trifluoromethanesulfonyl)imide anion; EC: ethylene carbonate; DEC: diethyl carbonate; FEC: fluoroethylene carbonate; VC: vinylene carbonate; DMC: dimethyl carbonate; EMC: ethyl methyl carbonate; DOL: 1,3-dioxolane; DME: 1,2-dimethoxyethane; LiNO_3_: lithium nitrate; PAA: poly(acrylic acid); PEO: poly(ethylene glycol)/poly(ethylene oxide); UPy: ureidopyrimidinone. ^a^ Self-healing (SH) conductive composite (SH polymer + carbon black (CB)) was melted at 100 °C and coated on a Si anode with a sharp blade. ^b^ Self-healing (SH) conductive composite (SH polymer + carbon black (CB)) was heated at 100 °C and drop cast onto a Si electrode to form a uniform coating. ^c^ Slurry (containing polymer binder, Si particles, conductive additive and, in some cases, a solvent) was applied on the current collector and dried. ^d^ In order to coat the current collector, polymer binder was dissolved in an appropriate solvent and spin coated. Subsequently, the solvent was removed by heating under vacuum. ^e^ A polymer mixture was degassed at room temperature and then coated onto an electrode using a sharp blade. ^f^ Monomers were dissolved in an appropriate solvent and coated onto a Cu foil by spin coating. After the removal of the solvent via heating, the desired protective coating was obtained. Subsequently, Li was deposited onto electrodes. ^g^ Coated Li foils were fabricated by the dip-coating method. ^h^ Polymer binder was dissolved in a solvent and subsequently dripped onto the surface of a Li-metal anode. After evaporation, the coated electrode was obtained.

## Data Availability

Not applicable.

## References

[1] Placke T., Kloepsch R., Dühnen S., Winter M. (2017). Lithium ion, lithium metal, and alternative rechargeable battery technologies: The odyssey for high energy density. J. Solid State Electrochem..

[2] Lopez J., Mackanic D.G., Cui Y., Bao Z. (2019). Designing polymers for advanced battery chemistries. Nat. Rev. Mater..

[3] Fichtner M., Edström K., Ayerbe E., Berecibar M., Bhowmik A., Castelli I.E., Clark S., Dominko R., Erakca M., Franco A.A. (2022). Rechargeable Batteries of the Future—The State of the Art from a BATTERY 2030+ Perspective. Adv. Energy Mater..

[4] Amici J., Asinari P., Ayerbe E., Barboux P., Bayle-Guillemaud P., Behm R.J., Berecibar M., Berg E., Bhowmik A., Bodoardo S. (2022). A Roadmap for Transforming Research to Invent the Batteries of the Future Designed within the European Large Scale Research Initiative BATTERY 2030+. Adv. Energy Mater..

[5] Li M., Lu J., Chen Z., Amine K. (2018). 30 Years of Lithium-Ion Batteries. Adv. Mater..

[6] Wu F., Maier J., Yu Y. (2020). Guidelines and trends for next-generation rechargeable lithium and lithium-ion batteries. Chem. Soc. Rev..

[7] Miao Y., Hynan P., von Jouanne A., Yokochi A. (2019). Current Li-Ion Battery Technologies in Electric Vehicles and Opportunities for Advancements. Energies.

[8] Forsyth M., Porcarelli L., Wang X., Goujon N., Mecerreyes D. (2019). Innovative Electrolytes Based on Ionic Liquids and Polymers for Next-Generation Solid-State Batteries. Acc. Chem. Res..

[9] Jin S., Mu D., Lu Z., Li R., Liu Z., Wang Y., Tian S., Dai C. (2022). A comprehensive review on the recycling of spent lithium-ion batteries: Urgent status and technology advances. J. Clean. Prod..

[10] Abu Nayem S.M., Ahmad A., Shaheen Shah S., Saeed Alzahrani A., Saleh Ahammad A.J., Aziz M.A. (2022). High Performance and Long-cycle Life Rechargeable Aluminum Ion Battery: Recent Progress, Perspectives and Challenges. Chem. Rec..

[11] Zhao Q., Stalin S., Zhao C.-Z., Archer L.A. (2020). Designing solid-state electrolytes for safe, energy-dense batteries. Nat. Rev. Mater..

[12] Li G., Huang B., Pan Z., Su X., Shao Z., An L. (2019). Advances in three-dimensional graphene-based materials: Configurations, preparation and application in secondary metal (Li, Na, K, Mg, Al)-ion batteries. Energy Environ. Sci..

[13] Abraham K.M. (2020). How Comparable Are Sodium-Ion Batteries to Lithium-Ion Counterparts?. ACS Energy Lett..

[14] Liu F., Wang T., Liu X., Fan L.-Z. (2021). Challenges and Recent Progress on Key Materials for Rechargeable Magnesium Batteries. Adv. Energy Mater..

[15] Anoopkumar V., Bibin J., Mercy T.D. (2020). Potassium-Ion Batteries: Key to Future Large-Scale Energy Storage?. ACS Appl. Energy Mater..

[16] Zheng S., Wang Q., Hou Y., Li L., Tao Z. (2021). Recent progress and strategies toward high performance zinc-organic batteries. J. Energy Chem..

[17] Ye T., Li L., Zhang Y. (2020). Recent Progress in Solid Electrolytes for Energy Storage Devices. Adv. Funct. Mater..

[18] Wang R., Cui W., Chu F., Wu F. (2020). Lithium metal anodes: Present and future. J. Energy Chem..

[19] Quartarone E., Mustarelli P. (2020). Review—Emerging Trends in the Design of Electrolytes for Lithium and Post-Lithium Batteries. J. Electrochem. Soc..

[20] Lee W., Muhammad S., Sergey C., Lee H., Yoon J., Kang Y.-M., Yoon W.-S. (2020). Advances in the Cathode Materials for Lithium Rechargeable Batteries. Angew. Chem. Int. Ed..

[21] Shi Y., Zhou X., Yu G. (2017). Material and Structural Design of Novel Binder Systems for High-Energy, High-Power Lithium-Ion Batteries. Acc. Chem. Res..

[22] Lee H., Yanilmaz M., Toprakci O., Fu K., Zhang X. (2014). A review of recent developments in membrane separators for rechargeable lithium-ion batteries. Energy Environ. Sci..

[23] Costa C.M., Lizundia E., Lanceros-Méndez S. (2020). Polymers for advanced lithium-ion batteries: State of the art and future needs on polymers for the different battery components. Prog. Energy Combust. Sci..

[24] Wang C., Wu H., Chen Z., McDowell M.T., Cui Y., Bao Z. (2013). Self-healing chemistry enables the stable operation of silicon microparticle anodes for high-energy lithium-ion batteries. Nat. Chem..

[25] Zhou D., Shanmukaraj D., Tkacheva A., Armand M., Wang G. (2019). Polymer Electrolytes for Lithium-Based Batteries: Advances and Prospects. Chem.

[26] Tarascon J.M., Armand M. (2001). Issues and challenges facing rechargeable lithium batteries. Nature.

[27] Lu D., Shao Y., Lozano T., Bennett W.D., Graff G.L., Polzin B., Zhang J., Engelhard M.H., Saenz N.T., Henderson W.A. (2015). Failure Mechanism for Fast-Charged Lithium Metal Batteries with Liquid Electrolytes. Adv. Energy Mater..

[28] Wang Q., Jiang L., Yu Y., Sun J. (2019). Progress of enhancing the safety of lithium ion battery from the electrolyte aspect. Nano Energy.

[29] Mai W., Yu Q., Han C., Kang F., Li B. (2020). Self-Healing Materials for Energy-Storage Devices. Adv. Funct. Mater..

[30] Mezzomo L., Ferrara C., Brugnetti G., Callegari D., Quartarone E., Mustarelli P., Ruffo R. (2020). Exploiting Self-Healing in Lithium Batteries: Strategies for Next-Generation Energy Storage Devices. Adv. Energy Mater..

[31] Döhler D., Michael P., Binder W.H., Binder W.H. (2013). Principles of Self Healing Polymers. Self Healing Polymers: From Principle to Application.

[32] Wang S., Urban M.W. (2020). Self-healing polymers. Nat. Rev. Mater..

[33] Zhao L., Sottos N.R. (2021). Autonomous Strategies for Improved Performance and Reliability of Li-Ion Batteries. Adv. Energy Mater..

[34] Ezeigwe E.R., Dong L., Manjunatha R., Tan M., Yan W., Zhang J. (2021). A review of self-healing electrode and electrolyte materials and their mitigating degradation of Lithium batteries. Nano Energy.

[35] Xu J., Ding C., Chen P., Tan L., Chen C., Fu J. (2020). Intrinsic self-healing polymers for advanced lithium-based batteries: Advances and strategies. Appl. Phys. Rev..

[36] Liao H., Zhong W., Li T., Han J., Sun X., Tong X., Zhang Y. (2022). A review of self-healing electrolyte and their applications in flexible/stretchable energy storage devices. Electrochim. Acta.

[37] Narayan R., Laberty-Robert C., Pelta J., Tarascon J.-M., Dominko R. (2022). Self-Healing: An Emerging Technology for Next-Generation Smart Batteries. Adv. Energy Mater..

[38] Cho S., Hwang S.Y., Oh D.X., Park J. (2021). Recent progress in self-healing polymers and hydrogels based on reversible dynamic B–O bonds: Boronic/boronate esters, borax, and benzoxaborole. J. Mater. Chem. A.

[39] Chan C.Y., Wang Z., Jia H., Ng P.F., Chow L., Fei B. (2021). Recent advances of hydrogel electrolytes in flexible energy storage devices. J. Mater. Chem. A.

[40] Bashir S., Hina M., Iqbal J., Rajpar A.H., Mujtaba M.A., Alghamdi N.A., Wageh S., Ramesh K., Ramesh S. (2020). Fundamental Concepts of Hydrogels: Synthesis, Properties, and Their Applications. Polymers.

[41] Wang Z., Li H., Tang Z., Liu Z., Ruan Z., Ma L., Yang Q., Wang D., Zhi C. (2018). Hydrogel Electrolytes for Flexible Aqueous Energy Storage Devices. Adv. Funct. Mater..

[42] Campanella A., Döhler D., Binder W.H. (2018). Self-Healing in Supramolecular Polymers. Macromol. Rapid Commun..

[43] Blaiszik B.J., Kramer S.L.B., Olugebefola S.C., Moore J.S., Sottos N.R., White S.R. (2010). Self-Healing Polymers and Composites. Annu. Rev. Mater. Res..

[44] Toohey K.S., Sottos N.R., Lewis J.A., Moore J.S., White S.R. (2007). Self-healing materials with microvascular networks. Nat. Mater..

[45] Huang S., Kong X., Xiong Y., Zhang X., Chen H., Jiang W., Niu Y., Xu W., Ren C. (2020). An overview of dynamic covalent bonds in polymer material and their applications. Eur. Polym. J..

[46] Guerre M., Taplan C., Winne J.M., Du Prez F.E. (2020). Vitrimers: Directing chemical reactivity to control material properties. Chem. Sci..

[47] Herbst F., Döhler D., Michael P., Binder W.H. (2013). Self-healing polymers via supramolecular forces. Macromol. Rapid Commun..

[48] Herbst F., Seiffert S., Binder W.H. (2012). Dynamic supramolecular poly(isobutylene)s for self-healing materials. Polym. Chem..

[49] Li C., Bhandary R., Marinow A., Ivanov D., Du M., Androsch R., Binder W.H. (2022). Synthesis and Characterization of Quadrupolar-Hydrogen-Bonded Polymeric Ionic Liquids for Potential Self-Healing Electrolytes. Polymers.

[50] Döhler D., Kang J., Cooper C.B., Tok J.B.H., Rupp H., Binder W.H., Bao Z. (2020). Tuning the Self-Healing Response of Poly(dimethylsiloxane)-Based Elastomers. ACS Appl. Polym. Mater..

[51] Shi Y., Wang M., Ma C., Wang Y., Li X., Yu G. (2015). A Conductive Self-Healing Hybrid Gel Enabled by Metal–Ligand Supramolecule and Nanostructured Conductive Polymer. Nano Lett..

[52] Guo P., Zhang H., Liu X., Sun J. (2018). Counteranion-Mediated Intrinsic Healing of Poly(ionic liquid) Copolymers. ACS Appl. Mater. Interfaces.

[53] Li X., Zhang H., Zhang P., Yu Y. (2018). A Sunlight-Degradable Autonomous Self-Healing Supramolecular Elastomer for Flexible Electronic Devices. Chem. Mater..

[54] Meurer J., Hniopek J., Ahner J., Schmitt M., Popp J., Zechel S., Peneva K., Hager M.D. (2021). In-depth characterization of self-healing polymers based on pi-pi nteractions. Beilstein J. Org. Chem..

[55] Fox J., Wie J.J., Greenland B.W., Burattini S., Hayes W., Colquhoun H.M., Mackay M.E., Rowan S.J. (2012). High-Strength, Healable, Supramolecular Polymer Nanocomposites. J. Am. Chem. Soc..

[56] Cao Y., Wu H., Allec S.I., Wong B.M., Nguyen D.-S., Wang C. (2018). A Highly Stretchy, Transparent Elastomer with the Capability to Automatically Self-Heal Underwater. Adv. Mater..

[57] Krishnakumar B., Sanka R.V.S.P., Binder W.H., Parthasarthy V., Rana S., Karak N. (2020). Vitrimers: Associative dynamic covalent adaptive networks in thermoset polymers. Chem. Eng. J..

[58] Reutenauer P., Buhler E., Boul P.J., Candau S.J., Lehn J.-M. (2009). Room Temperature Dynamic Polymers Based on Diels–Alder Chemistry. Chem. A Eur. J..

[59] Khan N.I., Halder S., Gunjan S.B., Prasad T. (2018). A review on Diels-Alder based self-healing polymer composites. IOP Conf. Ser. Mater. Sci. Eng..

[60] Kim S.-M., Jeon H., Shin S.-H., Park S.-A., Jegal J., Hwang S.Y., Oh D.X., Park J. (2018). Superior Toughness and Fast Self-Healing at Room Temperature Engineered by Transparent Elastomers. Adv. Mater..

[61] Irigoyen M., Fernández A., Ruiz A., Ruipérez F., Matxain J.M. (2019). Diselenide Bonds as an Alternative to Outperform the Efficiency of Disulfides in Self-Healing Materials. J. Org. Chem..

[62] Nishimura Y., Chung J., Muradyan H., Guan Z. (2017). Silyl Ether as a Robust and Thermally Stable Dynamic Covalent Motif for Malleable Polymer Design. J. Am. Chem. Soc..

[63] Chao A., Negulescu I., Zhang D. (2016). Dynamic Covalent Polymer Networks Based on Degenerative Imine Bond Exchange: Tuning the Malleability and Self-Healing Properties by Solvent. Macromolecules.

[64] Capelot M., Montarnal D., Tournilhac F., Leibler L. (2012). Metal-Catalyzed Transesterification for Healing and Assembling of Thermosets. J. Am. Chem. Soc..

[65] Zhang Z.P., Rong M.Z., Zhang M.Q. (2014). Room temperature self-healable epoxy elastomer with reversible alkoxyamines as crosslinkages. Polymer.

[66] Chen S., Wen K., Fan J., Bando Y., Golberg D. (2018). Progress and future prospects of high-voltage and high-safety electrolytes in advanced lithium batteries: From liquid to solid electrolytes. J. Mater. Chem. A.

[67] Mohtadi R. (2020). Beyond Typical Electrolytes for Energy Dense Batteries. Molecules.

[68] Osada I., de Vries H., Scrosati B., Passerini S. (2016). Ionic-Liquid-Based Polymer Electrolytes for Battery Applications. Angew. Chem. Int. Ed..

[69] Manthiram A., Yu X., Wang S. (2017). Lithium battery chemistries enabled by solid-state electrolytes. Nat. Rev. Mater..

[70] Wu N., Shi Y.-R., Lang S.-Y., Zhou J.-M., Liang J.-Y., Wang W., Tan S.-J., Yin Y.-X., Wen R., Guo Y.-G. (2019). Self-Healable Solid Polymeric Electrolytes for Stable and Flexible Lithium Metal Batteries. Angew. Chem. Int. Ed..

[71] Zhou B., Zuo C., Xiao Z., Zhou X., He D., Xie X., Xue Z. (2018). Self-Healing Polymer Electrolytes Formed via Dual-Networks: A New Strategy for Flexible Lithium Metal Batteries. Chem. A Eur. J..

[72] Zhou B., Yang M., Zuo C., Chen G., He D., Zhou X., Liu C., Xie X., Xue Z. (2020). Flexible, Self-Healing, and Fire-Resistant Polymer Electrolytes Fabricated via Photopolymerization for All-Solid-State Lithium Metal Batteries. ACS Macro Lett..

[73] Zhang L., Zhang P., Chang C., Guo W., Guo Z.H., Pu X. (2021). Self-Healing Solid Polymer Electrolyte for Room-Temperature Solid-State Lithium Metal Batteries. ACS Appl. Mater. Interfaces.

[74] Deng K., Zhou S., Xu Z., Xiao M., Meng Y. (2022). A high ion-conducting, self-healing and nonflammable polymer electrolyte with dynamic imine bonds for dendrite-free lithium metal batteries. Chem. Eng. J..

[75] Jo Y.H., Li S., Zuo C., Zhang Y., Gan H., Li S., Yu L., He D., Xie X., Xue Z. (2020). Self-Healing Solid Polymer Electrolyte Facilitated by a Dynamic Cross-Linked Polymer Matrix for Lithium-Ion Batteries. Macromolecules.

[76] Jo Y.H., Zhou B., Jiang K., Li S., Zuo C., Gan H., He D., Zhou X., Xue Z. (2019). Self-healing and shape-memory solid polymer electrolytes with high mechanical strength facilitated by a poly(vinyl alcohol) matrix. Polym. Chem..

[77] Li R., Fang Z., Wang C., Zhu X., Fu X., Fu J., Yan W., Yang Y. (2022). Six-armed and dicationic polymeric ionic liquid for highly stretchable, nonflammable and notch-insensitive intrinsic self-healing solid-state polymer electrolyte for flexible and safe lithium batteries. Chem. Eng. J..

[78] Cao X., Zhang P., Guo N., Tong Y., Xu Q., Zhou D., Feng Z. (2021). Self-healing solid polymer electrolyte based on imine bonds for high safety and stable lithium metal batteries. RSC Adv..

[79] Zhou B., He D., Hu J., Ye Y., Peng H., Zhou X., Xie X., Xue Z. (2018). A flexible, self-healing and highly stretchable polymer electrolyte via quadruple hydrogen bonding for lithium-ion batteries. J. Mater. Chem. A.

[80] Gu W., Li F., Liu T., Gong S., Gao Q., Li J., Fang Z. (2022). Recyclable, Self-Healing Solid Polymer Electrolytes by Soy Protein-Based Dynamic Network. Adv. Sci..

[81] Guo C., Cao Y., Li J., Li H., Kumar Arumugam S., Oleksandr S., Chen F. (2022). Solvent-free green synthesis of nonflammable and self-healing polymer film electrolytes for lithium metal batteries. Appl. Energy.

[82] Sun S., Wu T. (2022). Preparation and properties of self-healable solid-state polymer electrolytes based on covalent adaptive networks enabled by disulfide bond. J. Polym. Sci..

[83] Sun Z., Wu J., Yuan H., Lan J., Yu Y., Zhu Y., Yang X. (2022). Self-healing polymer electrolyte for long-life and recyclable lithium-metal batteries. Mater. Today Energy.

[84] Wang H., Huang Y., Shi Z., Zhou X., Xue Z. (2022). Disulfide Metathesis-Assisted Lithium-Ion Conduction for PEO-Based Polymer Electrolytes. ACS Macro Lett..

[85] Xue X., Cao X., Wan L., Tong Y., Li T., Xie Y. (2022). Crosslinked network solid polymer electrolyte with self-healing ability and high stability for lithium metal battery. Polym. Int..

[86] Zhang L.-J., Zhou L., Yan Y., Wu M.-X., Wu N. (2022). Fast self-healing solid polymer electrolyte with high ionic conductivity for lithium metal batteries. New J. Chem..

[87] Zhou S., Deng K., Xu Z., Xiao M., Meng Y. (2022). Highly conductive self-healing polymer electrolytes based on synergetic dynamic bonds for highly safe lithium metal batteries. Chem. Eng. J..

[88] Jing B.B., Evans C.M. (2019). Catalyst-Free Dynamic Networks for Recyclable, Self-Healing Solid Polymer Electrolytes. J. Am. Chem. Soc..

[89] Zhu X., Fang Z., Deng Q., Zhou Y., Fu X., Wu L., Yan W., Yang Y. (2022). Poly(ionic liquid)@PEGMA Block Polymer Initiated Microphase Separation Architecture in Poly(ethylene oxide)-Based Solid-State Polymer Electrolyte for Flexible and Self-Healing Lithium Batteries. ACS Sustain. Chem. Eng..

[90] Gan H., Zhang Y., Li S., Yu L., Wang J., Xue Z. (2021). Self-Healing Single-Ion Conducting Polymer Electrolyte Formed via Supramolecular Networks for Lithium Metal Batteries. ACS Appl. Energy Mater..

[91] Jaumaux P., Liu Q., Zhou D., Xu X., Wang T., Wang Y., Kang F., Li B., Wang G. (2020). Deep-Eutectic-Solvent-Based Self-Healing Polymer Electrolyte for Safe and Long-Life Lithium-Metal Batteries. Angew. Chem. Int. Ed..

[92] Chen T., Kong W., Zhang Z., Wang L., Hu Y., Zhu G., Chen R., Ma L., Yan W., Wang Y. (2018). Ionic liquid-immobilized polymer gel electrolyte with self-healing capability, high ionic conductivity and heat resistance for dendrite-free lithium metal batteries. Nano Energy.

[93] Wang C., Li R., Chen P., Fu Y., Ma X., Shen T., Zhou B., Chen K., Fu J., Bao X. (2021). Highly stretchable, non-flammable and notch-insensitive intrinsic self-healing solid-state polymer electrolyte for stable and safe flexible lithium batteries. J. Mater. Chem. A.

[94] Tang Z., Lyu X., Xiao A., Shen Z., Fan X. (2018). High-Performance Double-Network Ion Gels with Fast Thermal Healing Capability via Dynamic Covalent Bonds. Chem. Mater..

[95] Guo P., Su A., Wei Y., Liu X., Li Y., Guo F., Li J., Hu Z., Sun J. (2019). Healable, Highly Conductive, Flexible, and Nonflammable Supramolecular Ionogel Electrolytes for Lithium-Ion Batteries. ACS Appl. Mater. Interfaces.

[96] Tian X., Yang P., Yi Y., Liu P., Wang T., Shu C., Qu L., Tang W., Zhang Y., Li M. (2020). Self-healing and high stretchable polymer electrolytes based on ionic bonds with high conductivity for lithium batteries. J. Power Sources.

[97] D’Angelo A.J., Panzer M.J. (2019). Design of Stretchable and Self-Healing Gel Electrolytes via Fully Zwitterionic Polymer Networks in Solvate Ionic Liquids for Li-Based Batteries. Chem. Mater..

[98] Li F., Nguyen G.T.M., Vancaeyzeele C., Vidal F., Plesse C. (2022). Healable Ionoelastomer Designed from Polymeric Ionic Liquid and Vitrimer Chemistry. ACS Appl. Polym. Mater..

[99] Wan L., Cao X., Xue X., Tong Y., Ci S., Huang H., Zhou D. (2022). Self-Healing and Flexible Ionic Gel Polymer Electrolyte Based on Reversible Bond for High-Performance Lithium Metal Batteries. Energy Technol..

[100] Chen X., Yi L., Zou C., Liu J., Yu J., Zang Z., Tao X., Luo Z., Guo X., Chen G. (2022). High-Performance Gel Polymer Electrolyte with Self-Healing Capability for Lithium-Ion Batteries. ACS Appl. Energy Mater..

[101] Xia S., Lopez J., Liang C., Zhang Z., Bao Z., Cui Y., Liu W. (2019). High-Rate and Large-Capacity Lithium Metal Anode Enabled by Volume Conformal and Self-Healable Composite Polymer Electrolyte. Adv. Sci..

[102] Zhou B., Jo Y.H., Wang R., He D., Zhou X., Xie X., Xue Z. (2019). Self-healing composite polymer electrolyte formed via supramolecular networks for high-performance lithium-ion batteries. J. Mater. Chem. A.

[103] Whiteley J.M., Taynton P., Zhang W., Lee S.-H. (2015). Ultra-thin Solid-State Li-Ion Electrolyte Membrane Facilitated by a Self-Healing Polymer Matrix. Adv. Mater..

[104] Whiteley J.M., Hafner S., Zhu C., Zhang W., Lee S.-H. (2017). Stable Lithium Deposition Using a Self-Optimizing Solid Electrolyte Composite. J. Electrochem. Soc..

[105] Katcharava Z., Marinow A., Bhandary R., Binder W.H. (2022). 3D Printable Composite Polymer Electrolytes: Influence of SiO2 Nanoparticles on 3D-Printability. Nanomaterials.

[106] Li J., Yang L., Zhang H., Ji X. (2022). Self-healing composite solid electrolytes with enhanced Li+ transport and mechanical properties for safe lithium metal batteries. Chem. Eng. J..

[107] Varzi A., Raccichini R., Passerini S., Scrosati B. (2016). Challenges and prospects of the role of solid electrolytes in the revitalization of lithium metal batteries. J. Mater. Chem. A.

[108] Xie Z., Hu B.-L., Li R.-W., Zhang Q. (2021). Hydrogen Bonding in Self-Healing Elastomers. ACS Omega.

[109] Huang X., Nakagawa S., Houjou H., Yoshie N. (2021). Insights into the Role of Hydrogen Bonds on the Mechanical Properties of Polymer Networks. Macromolecules.

[110] Song Y., Chen Y., Chen R., Zhang H., Shi D., Wang Y., Dong W., Ma P., Zhao Y. (2021). Use of Quadruple Hydrogen Bonding as the Switching Phase in Thermo- and Light-Responsive Shape Memory Hydrogel. ACS Appl. Polym. Mater..

[111] Lugger S.J.D., Houben S.J.A., Foelen Y., Debije M.G., Schenning A.P.H.J., Mulder D.J. (2022). Hydrogen-Bonded Supramolecular Liquid Crystal Polymers: Smart Materials with Stimuli-Responsive, Self-Healing, and Recyclable Properties. Chem. Rev..

[112] Chen S., Mahmood N., Beiner M., Binder W.H. (2015). Self-Healing Materials from V- and H-Shaped Supramolecular Architectures. Angew. Chem. Int. Ed..

[113] Watanabe M., Thomas M.L., Zhang S., Ueno K., Yasuda T., Dokko K. (2017). Application of Ionic Liquids to Energy Storage and Conversion Materials and Devices. Chem. Rev..

[114] Galiński M., Lewandowski A., Stępniak I. (2006). Ionic liquids as electrolytes. Electrochim. Acta.

[115] Gao Y.-R., Cao J.-F., Shu Y., Wang J.-H. (2021). Research progress of ionic liquids-based gels in energy storage, sensors and antibacterial. Green Chem. Eng..

[116] Zhang W., Jiang H., Chang Z., Wu W., Wu G., Wu R., Li J. (2020). Recent achievements in self-healing materials based on ionic liquids: A review. J. Mater. Sci..

[117] Yang G., Song Y., Wang Q., Zhang L., Deng L. (2020). Review of ionic liquids containing, polymer/inorganic hybrid electrolytes for lithium metal batteries. Mater. Des..

[118] Eshetu G.G., Mecerreyes D., Forsyth M., Zhang H., Armand M. (2019). Polymeric ionic liquids for lithium-based rechargeable batteries. Mol. Syst. Des. Eng..

[119] McGrath L.M., Rohan J.F. (2020). Pyrrolidinium Containing Ionic Liquid Electrolytes for Li-Based Batteries. Molecules.

[120] Baskoro F., Wong H.Q., Yen H.-J. (2019). Strategic Structural Design of a Gel Polymer Electrolyte toward a High Efficiency Lithium-Ion Battery. ACS Appl. Energy Mater..

[121] Liang S., Yan W., Wu X., Zhang Y., Zhu Y., Wang H., Wu Y. (2018). Gel polymer electrolytes for lithium ion batteries: Fabrication, characterization and performance. Solid State Ion..

[122] Tamate R., Watanabe M. (2020). Recent progress in self-healable ion gels. Sci. Technol. Adv. Mater..

[123] Boaretto N., Meabe L., Martinez-Ibañez M., Armand M., Zhang H. (2020). Review—Polymer Electrolytes for Rechargeable Batteries: From Nanocomposite to Nanohybrid. J. Electrochem. Soc..

[124] Yao P., Yu H., Ding Z., Liu Y., Lu J., Lavorgna M., Wu J., Liu X. (2019). Review on Polymer-Based Composite Electrolytes for Lithium Batteries. Front. Chem..

[125] Zhang L., Al-Mamun M. (2022). Investigating ionic liquids for optimizing lithium metal anode. Green Energy Environ..

[126] Rupp H., Bhandary R., Kulkarni A., Binder W. (2022). Printable Electrolytes: Tuning 3D-Printing by Multiple Hydrogen Bonds and Added Inorganic Lithium-Salts. Adv. Mater. Technol..

[127] Rupp H., Binder W.H. (2021). 3D Printing of Solvent-Free Supramolecular Polymers. Front. Chem..

[128] Zhang J., Zhao J., Yue L., Wang Q., Chai J., Liu Z., Zhou X., Li H., Guo Y., Cui G. (2015). Safety-Reinforced Poly(Propylene Carbonate)-Based All-Solid-State Polymer Electrolyte for Ambient-Temperature Solid Polymer Lithium Batteries. Adv. Energy Mater..

[129] Fan X., Chen L., Ji X., Deng T., Hou S., Chen J., Zheng J., Wang F., Jiang J., Xu K. (2018). Highly Fluorinated Interphases Enable High-Voltage Li-Metal Batteries. Chem.

[130] Liu K., Pei A., Lee H.R., Kong B., Liu N., Lin D., Liu Y., Liu C., Hsu P.-c., Bao Z. (2017). Lithium Metal Anodes with an Adaptive “Solid-Liquid” Interfacial Protective Layer. J. Am. Chem. Soc..

[131] Li N.W., Shi Y., Yin Y.X., Zeng X.X., Li J.Y., Li C.J., Wan L.J., Wen R., Guo Y.G. (2018). A Flexible Solid Electrolyte Interphase Layer for Long-Life Lithium Metal Anodes. Angew. Chem. Int. Ed..

[132] Zhu B., Wang X., Yao P., Li J., Zhu J. (2019). Towards high energy density lithium battery anodes: Silicon and lithium. Chem. Sci..

[133] Krauskopf T., Richter F.H., Zeier W.G., Janek J. (2020). Physicochemical Concepts of the Lithium Metal Anode in Solid-State Batteries. Chem. Rev..

[134] Hu Z., Li J., Zhang X., Zhu Y. (2020). Strategies to Improve the Performance of Li Metal Anode for Rechargeable Batteries. Front. Chem..

[135] Higgins T.M., Park S.-H., King P.J., Zhang C., McEvoy N., Berner N.C., Daly D., Shmeliov A., Khan U., Duesberg G. (2016). A Commercial Conducting Polymer as Both Binder and Conductive Additive for Silicon Nanoparticle-Based Lithium-Ion Battery Negative Electrodes. ACS Nano.

[136] Casimir A., Zhang H., Ogoke O., Amine J.C., Lu J., Wu G. (2016). Silicon-based anodes for lithium-ion batteries: Effectiveness of materials synthesis and electrode preparation. Nano Energy.

[137] Chen Z., Wang C., Lopez J., Lu Z., Cui Y., Bao Z. (2015). High-Areal-Capacity Silicon Electrodes with Low-Cost Silicon Particles Based on Spatial Control of Self-Healing Binder. Adv. Energy Mater..

[138] Lopez J., Chen Z., Wang C., Andrews S.C., Cui Y., Bao Z. (2016). The Effects of Cross-Linking in a Supramolecular Binder on Cycle Life in Silicon Microparticle Anodes. ACS Appl. Mater. Interfaces.

[139] Munaoka T., Yan X., Lopez J., To J.W.F., Park J., Tok J.B.-H., Cui Y., Bao Z. (2018). Ionically Conductive Self-Healing Binder for Low Cost Si Microparticles Anodes in Li-Ion Batteries. Adv. Energy Mater..

[140] Kim D., Hyun S., Han S.M. (2018). Freestanding silicon microparticle and self-healing polymer composite design for effective lithiation stress relaxation. J. Mater. Chem. A.

[141] Zhang G., Yang Y., Chen Y., Huang J., Zhang T., Zeng H., Wang C., Liu G., Deng Y. (2018). A Quadruple-Hydrogen-Bonded Supramolecular Binder for High-Performance Silicon Anodes in Lithium-Ion Batteries. Small.

[142] Xu Z., Yang J., Zhang T., Nuli Y., Wang J., Hirano S.-i. (2018). Silicon Microparticle Anodes with Self-Healing Multiple Network Binder. Joule.

[143] Yang J., Zhang L., Zhang T., Wang X., Gao Y., Fang Q. (2018). Self-healing strategy for Si nanoparticles towards practical application as anode materials for Li-ion batteries. Electrochem. Commun..

[144] Ryu J., Kim S., Kim J., Park S., Lee S., Yoo S., Kim J., Choi N.-S., Ryu J.-H., Park S. (2020). Room-Temperature Crosslinkable Natural Polymer Binder for High-Rate and Stable Silicon Anodes. Adv. Funct. Mater..

[145] Jeong Y.K., Choi J.W. (2019). Mussel-Inspired Self-Healing Metallopolymers for Silicon Nanoparticle Anodes. ACS Nano.

[146] Gupta A., Badam R., Matsumi N. (2022). Heavy-Duty Performance from Silicon Anodes Using Poly(BIAN)/Poly(acrylic acid)-Based Self-Healing Composite Binder in Lithium-Ion Secondary Batteries. ACS Appl. Energy Mater..

[147] Jiang M., Mu P., Zhang H., Dong T., Tang B., Qiu H., Chen Z., Cui G. (2022). An Endotenon Sheath-Inspired Double-Network Binder Enables Superior Cycling Performance of Silicon Electrodes. Nano-Micro Lett..

[148] Nam J., Jang W., Rajeev K.K., Lee J.-H., Kim Y., Kim T.-H. (2021). Ion-conductive self-healing polymer network based on reversible imine bonding for Si electrodes. J. Power Sources.

[149] Hu L., Zhang X., Zhao P., Fan H., Zhang Z., Deng J., Ungar G., Song J. (2021). Gradient H-Bonding Binder Enables Stable High-Areal-Capacity Si-Based Anodes in Pouch Cells. Adv. Mater..

[150] Nam J., Kim E., K.K R., Kim Y., Kim T.-H. (2020). A conductive self healing polymeric binder using hydrogen bonding for Si anodes in lithium ion batteries. Sci. Rep..

[151] K.K R., Nam J., Kim E., Kim Y., Kim T.-H. (2020). A self-healable polymer binder for Si anodes based on reversible Diels–Alder chemistry. Electrochim. Acta.

[152] Zheng G., Wang C., Pei A., Lopez J., Shi F., Chen Z., Sendek A.D., Lee H.-W., Lu Z., Schneider H. (2016). High-Performance Lithium Metal Negative Electrode with a Soft and Flowable Polymer Coating. ACS Energy Lett..

[153] Cui X., Chu Y., Qin L., Pan Q. (2018). Stabilizing Li Metal Anodes through a Novel Self-Healing Strategy. ACS Sustain. Chem. Eng..

[154] Yu Z., Mackanic D.G., Michaels W., Lee M., Pei A., Feng D., Zhang Q., Tsao Y., Amanchukwu C.V., Yan X. (2019). A Dynamic, Electrolyte-Blocking, and Single-Ion-Conductive Network for Stable Lithium-Metal Anodes. Joule.

[155] Wang G., Chen C., Chen Y., Kang X., Yang C., Wang F., Liu Y., Xiong X. (2020). Self-Stabilized and Strongly Adhesive Supramolecular Polymer Protective Layer Enables Ultrahigh-Rate and Large-Capacity Lithium-Metal Anode. Angew. Chem. Int. Ed..

[156] Cui X., Chu Y., Wang X., Zhang X., Li Y., Pan Q. (2021). Stabilizing Lithium Metal Anodes by a Self-Healable and Li-Regulating Interlayer. ACS Appl. Mater. Interfaces.

[157] Beaulieu L.Y., Eberman K.W., Turner R.L., Krause L.J., Dahn J.R. (2001). Colossal Reversible Volume Changes in Lithium Alloys. Electrochem. Solid-State Lett..

[158] Liu N., Lu Z., Zhao J., McDowell M.T., Lee H.-W., Zhao W., Cui Y. (2014). A pomegranate-inspired nanoscale design for large-volume-change lithium battery anodes. Nat. Nanotechnol..

[159] Kwon T.-w., Choi J.W., Coskun A. (2018). The emerging era of supramolecular polymeric binders in silicon anodes. Chem. Soc. Rev..

[160] Liu X.H., Zhong L., Huang S., Mao S.X., Zhu T., Huang J.Y. (2012). Size-Dependent Fracture of Silicon Nanoparticles During Lithiation. ACS Nano.

[161] Ma Z., Gao X., Wang Y., Lu C. (2016). Effects of size and concentration on diffusion-induced stress in lithium-ion batteries. J. Appl. Phys..

[162] Zhou X., Yin Y.-X., Wan L.-J., Guo Y.-G. (2012). Self-Assembled Nanocomposite of Silicon Nanoparticles Encapsulated in Graphene through Electrostatic Attraction for Lithium-Ion Batteries. Adv. Energy Mater..

[163] Li H., Huang X., Chen L., Zhou G., Zhang Z., Yu D., Jun Mo Y., Pei N. (2000). The crystal structural evolution of nano-Si anode caused by lithium insertion and extraction at room temperature. Solid State Ion..

[164] Cordier P., Tournilhac F., Soulié-Ziakovic C., Leibler L. (2008). Self-healing and thermoreversible rubber from supramolecular assembly. Nature.

[165] Goodenough J.B., Kim Y. (2010). Challenges for Rechargeable Li Batteries. Chem. Mater..

[166] Cheng X.-B., Zhang R., Zhao C.-Z., Zhang Q. (2017). Toward Safe Lithium Metal Anode in Rechargeable Batteries: A Review. Chem. Rev..

